# MR-MAS: an end-to-end multi-agent system for autonomous Mendelian randomization analysis

**DOI:** 10.1093/bioinformatics/btag722

**Published:** 2026-10-20

**Authors:** Wendi Wei, Yuling Fan, Lin Li, Thuc Duy Le, Xiaomei Li, Debo Cheng, Zaiwen Feng

**Affiliations:** College of Informatics, Huazhong Agricultural University, Wuhan, 430070, China; College of Informatics, Huazhong Agricultural University, Wuhan, 430070, China; College of Plant Science & Technology, Huazhong Agricultural University, Wuhan, 430070, China; College of Engineering and Information Technology, Adelaide University, Adelaide, SA 5095, Australia; Business Banking, Commonwealth Bank, Sydney, NSW 2000, Australia; School of Computer Science and Technology, Hainan University, Haikou, 570228, China; College of Informatics, Huazhong Agricultural University, Wuhan, 430070, China; Hubei Key Laboratory of Agricultural Bioinformatics, Huazhong Agricultural University, Wuhan, 430070, China

## Abstract

**Motivation:**

Understanding disease-related causal mechanisms is essential for biomedical research, yet the practical use of Mendelian Randomization (MR) remains constrained by fragmented analytical workflows and a strong dependence on specialized expertise. These challenges limit the efficient, standardized, and reproducible execution of end-to-end MR analyses.

**Results:**

We propose MR-MAS, an end-to-end automated MR analysis system built on a multi-agent architecture. MR-MAS leverages the reasoning and coordination capabilities of large language models (LLMs) and integrates external tools to unify data acquisition, causal effect estimation, results interpretation, and manuscript preparation into a single workflow, thereby reducing manual effort. MR-MAS further incorporates a structured Mendelian Randomization Knowledge Base (MRKB) to support MR method implementation and targeted repair of MR analysis code. Across diverse test scenarios, MR-MAS demonstrated stable workflow execution, reproduced several literature-supported causal signals, and maintained consistent causal estimates across repeated runs and different model backends. Additional evaluations showed robust trait retrieval and improved code-repair performance with MRKB guidance. These results suggest that MR-MAS may provide practical support for scalable, standardized, and reproducible MR analyses in biomedical research.

**Availability and Implementation:**

The source code, MRKB, and benchmark data are freely available at https://github.com/Zaiwen/MR-MAS, with the version used in this study archived at https://doi.org/10.5281/zenodo.20342636.

## 1 Introduction

In biomedical research, accurately identifying causal relationships between traits, such as risk factors, lifestyle behaviours, or biomarkers, and diseases is fundamental for understanding disease mechanisms and designing effective interventions (Russo and Williamson 2007). However, traditional observational studies are often affected by unmeasured confounding and reverse causation (Greenland *et al.* 1999, Lawlor *et al.* 2008, Toonsi *et al.* 2024). For example, the association between smoking and impaired lung function may be partly driven by socioeconomic factors, and elevated levels of certain biomarkers may simply reflect disease progression. These sources of bias limit the reliability of observational studies for causal inference.

Mendelian Randomization (MR) offers a powerful alternative by leveraging the random allocation of genetic variants and using them as instrumental variables for causal inference (Smith and Ebrahim 2003). With the availability of large-scale genomic resources such as the UK Biobank (Bycroft *et al.* 2018), OpenGWAS (Elsworth *et al.* 2020), and the GWAS Catalog (Buniello *et al.* 2019), MR has been widely adopted to elucidate causal relationships, identify actionable risk factors and prioritize therapeutic targets.

Despite substantial methodological progress, practical MR workflows remain highly fragmented and difficult to execute reproducibly. A full analysis typically involves multiple steps, including GWAS data retrieval, instrument selection, causal effect estimation, sensitivity analyses, and result interpretation (Lawlor *et al.* 2008, Hemani *et al.* 2018), often requiring frequent switches between different tools and platforms. This fragmentation increases the likelihood of human error and imposes substantial barriers for clinicians and researchers without strong computational or statistical expertise. These challenges highlight the need for automated, standardized and reproducible MR workflows.

In recent years, large language models (LLMs) have shown substantial potential for building ‘AI scientists’ through their capabilities in natural language processing, reasoning, code generation and task planning (Achiam *et al.* 2023, Chang *et al.* 2024). A growing body of work has demonstrated that LLM-driven agent systems (Wei *et al.* 2022, Yao *et al.* 2023) can perform complex scientific tasks via role specialization and collaborative reasoning. For example, Agent Laboratory (Schmidgall *et al.* 2025) presents a multi-agent system that autonomously conducts literature retrieval, data processing, experimental analysis and scientific writing, demonstrating the feasibility of using AI as a research assistant. AI Scientist-v2 (Yamada *et al.* 2025) constructs a fully automated framework that spans hypothesis generation, experiment design and execution, result analysis and manuscript writing, and further advances the ‘AI scientists’ paradigm by incorporating a multi-agent collaboration mechanism guided by tree search. ChemCrow (Bran *et al.* 2024) tightly integrates LLMs with a chemistry toolset to achieve automated reaction planning and experimental task execution. Virtual Lab (Swanson *et al.* 2025) further demonstrates the innovative capacity of agent systems in real biomedical scenarios, where newly designed SARS-CoV-2 nanobodies exhibit strong performance in experimental validation.

Agent-based systems have also shown broad applicability across multiple areas of the life sciences (Yang and Velazquez-Villarreal 2025). For instance, CRISPR-GPT (Qu *et al.* 2025) can autonomously plan and analyse gene-editing experiments, SciAgents (Ghafarollahi and Buehler 2025) combines multiple agents with knowledge-graph reasoning to generate hypotheses and predict material properties, and AutoBA (Zhou *et al.* 2024) leverages large language models to generate data analysis pipelines, perform cross-tool reasoning, and produce interpretable reports for multi-omics analyses. Biomni (Huang *et al.* 2025) further demonstrates the potential of general-purpose biomedical agents by integrating diverse biomedical tools and databases to support a broad range of research tasks. Collectively, these studies suggest that multi-agent systems are well suited to scientific tasks requiring multi-source data integration, multi-step reasoning, and coordination across tools.

In the context of automated MR research, MRAgent (Xu *et al.* 2025) focuses on automatically generating candidate exposure–outcome pairs related to a given disease from the literature and linking them to downstream MR analyses and report generation. However, the set of MR methods supported by MRAgent remains limited in scope, which constrains its ability to support more advanced and robust approaches for addressing pleiotropy and invalid instrumental variables. In addition, the generated reports primarily consist of simple summaries with limited informational content. The outputs of MRAgent do not include executable code or complete records of parameters and processing procedures, thereby restricting reproducibility. Consequently, further improvements in robustness, scalability, and process transparency are still required for automated MR systems to be effective in real-world research settings.

To address these challenges, we propose MR-MAS, an end-to-end multi-agent framework for automating MR analysis in biomedical research. MR-MAS organizes the MR research process into a coordinated workflow that spans research plan formulation, GWAS data preparation, causal effect estimation, result interpretation, and manuscript drafting. Rather than treating these steps as separate manual procedures, MR-MAS assigns them to role-specialized agents that interact with shared memory, external data resources, and local computational tools. In doing so, it produces traceable analytical artefacts, including standardized datasets, executable analysis scripts, statistical outputs, and manuscript drafts, thereby supporting workflow transparency and reproducibility. To enhance robustness during the causal effect estimation stage, MR-MAS further incorporates a structured Mendelian Randomization Knowledge Base (MRKB) to support method-aware code validation and targeted error detection and repair.

The main contributions of this work are summarized as follows.

- We design MR-MAS, an end-to-end automated MR analysis system driven by multi-agent collaboration, integrating GWAS data retrieval and processing, MR analysis, result interpretation, and manuscript drafting into a unified workflow. This architecture supports a largely automated MR workflow and lowers the technical barrier to MR analysis.
- We develop an extensible MR Knowledge Base (MRKB) that organizes MR methods and related computational resources in a structured YAML (Yet Another Markup Language) format. MRKB supports method-aware error diagnosis and targeted code repair, improving the robustness of MR analysis execution.
- We systematically evaluate MR-MAS in terms of analytical consistency, efficiency, robustness, and reproducibility. The results suggest its potential to support scalable and reproducible automated MR analyses.

## 2 Methods

### 2.1 Problem formulation

In MR-MAS, automated MR analysis is formulated as a stage-wise task planning and execution problem. Given a user-defined MR research objective G, the system first parses the exposure trait, outcome trait, and optional analysis specifications. The goal is to automatically execute an end-to-end MR workflow and produce structured research artefacts.

The workflow is decomposed into an ordered sequence of stages, denoted as $S=[s_{1},s_{2},\ldots ,s_{n}]$, where $s_{i}$ denotes the *i*-th MR analysis stage. In this study, the default workflow consists of six stages: plan formulation, data preparation, MR analysis, result interpretation, manuscript drafting, and manuscript review. Each stage takes upstream outputs and shared contextual information as input, invokes the corresponding computational or retrieval tools, and generates structured intermediate artefacts for subsequent stages.

### 2.2 Framework overview

As illustrated in Fig. 1, MR-MAS consists of five core components: (i) Six role-specialized LLM agents: Biological Engineer, Statistical Geneticist, Algorithm Engineer, Data Analyst, Writing Specialist, and Reviewer, collaborate to execute different stages of the MR workflow; (ii) Shared Memory: Maintains validated cross-stage artefacts and temporary stage-level information to preserve contextual continuity; (iii) Tool Pool: Provides access to external resources and local computing environments; (iv) MR Knowledge Base (MRKB): Organizes structured knowledge of MR methods and R package functions to support code error repair; and (v) Validation and Repair Mechanism: Evaluates stage outputs and determines whether the workflow should proceed, retry, repair, or stop.

**Figure 1:**
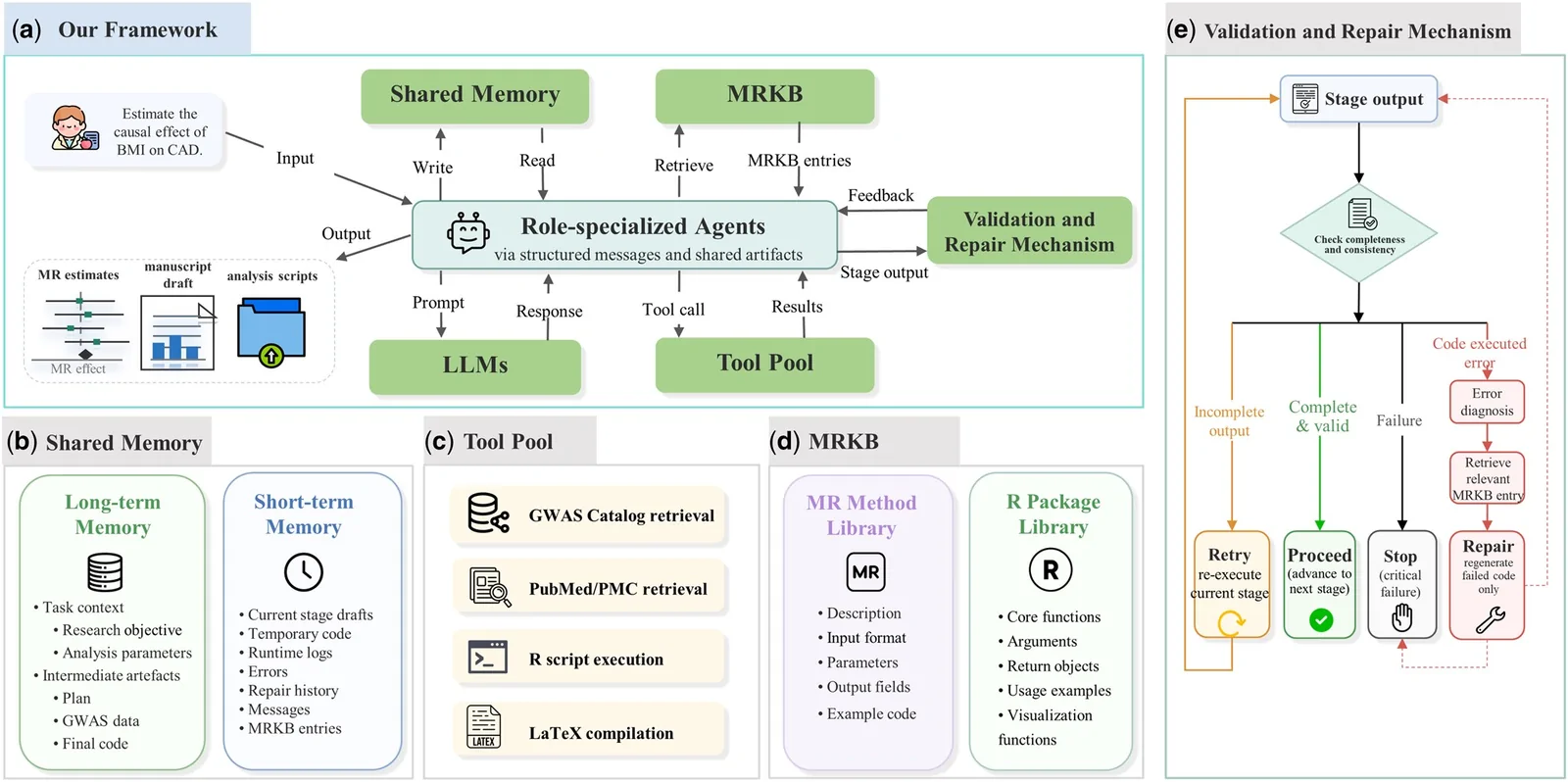
Overview of MR-MAS, the proposed multi-agent framework for automated MR analysis. (a) MR-MAS consists of five core components: role-specialized LLM agents, shared memory, tool pool, MRKB, and a validation and repair mechanism. (b) Shared memory supports long-term artefacts storage and short-term stage-specific context management. (c) Tool pool provides access to GWAS Catalog retrieval, PubMed/PMC retrieval, R execution, and LaTeX compilation. (d) MRKB stores structured knowledge of MR methods and R packages. (e) Validation and repair mechanism enables stage-level proceed, retry, repair, or stop decisions.

Given a user-defined MR research objective, MR-MAS decomposes the task into stage-wise subtasks, activates the corresponding agents, retrieves external data and methodological knowledge when needed, and validates stage outputs before passing them to downstream stages. The following subsections describe the implementation of these components in more detail.

### 2.3 Stage-wise multi-agent workflow execution

During execution, each workflow stage is coordinated by designated role-specific agents.

For a given stage $s_{i}$, the system activates the corresponding agent according to the current stage task and provides the user-defined research objective and shared memory. This process can be represented as $o_{i}=A_{i}(p_{i},s_{i},G,M)$, where $A_{i}$ denotes the role-specialized agent responsible for stage $s_{i}$, $p_{i}$ denotes the corresponding agent prompt, *G* denotes the user-defined MR research objective, *M* denotes the shared memory, and $o_{i}$ denotes the output artefacts generated by this stage. The detailed structure of shared memory is described in the Section 2.4 Section.

Information transfer among agents is implemented through a stage-wise communication protocol based on shared memory. At each stage, the active agent receives a communication context related to its role and the current stage, including the research objective, current stage information, available stage artefacts, short-term interaction history, stage-specific notes, and feedback from previous steps. Based on this context, the agent generates the output required for the current stage.

The generated output is stored in short-term history together with the corresponding stage information, step index, and feedback. Once the output passes stage-level validation, it is further written into long-term memory and used as input for subsequent steps or downstream stages. When subsequent agents are executed, they retrieve the validated artefacts and relevant short-term contextual information from shared memory, thereby enabling information transfer across agents and stages.

### 2.4 Shared memory

To preserve contextual continuity across long, multi-stage MR analyses, the framework maintains a shared memory *M*, which is divided into long-term memory and short-term memory.

Long-term memory stores validated information that can be reliably reused by downstream stages, including task context and key intermediate artefacts. The task context records the user-defined research objective and analysis parameters, whereas key intermediate artefacts include the research plan, harmonized GWAS datasets, MR analysis scripts, MR execution logs, and manuscript drafts. The contents of long-term memory serve as validated inputs for subsequent data analysis, result interpretation, and manuscript generation.

Short-term memory stores temporary information generated within the current stage, including stage drafts, temporary code, runtime errors, intermediate messages, repair records, and MRKB method or tool descriptions retrieved for that stage. It primarily supports within-stage iterative execution and error repair. Once the stage output passes validation, necessary results are written into long-term memory, whereas temporary information and stage-specific MRKB retrieval snippets are cleaned according to the history length constraint and expiration mechanism to reduce contextual redundancy.

### 2.5 Tool pool

To support the automated execution of an end-to-end MR research workflow, the system integrates external biomedical resources with local computing environments. Specifically, it provides programmatic access to the GWAS Catalog for GWAS study retrieval and summary statistics download, and supports PubMed (Canese and Weis 2013) and PubMed Central retrieval for literature evidence collection. Local R and LaTeX environments are employed to execute MR analysis scripts and compile manuscript drafts, respectively. Together, these components provide the computational and retrieval infrastructure required for the automated workflow.

### 2.6 MR Knowledge Base

The MR Knowledge Base (MRKB) is an extensible YAML-based knowledge base for organizing MR methodological and computational resources. It contains two components: the MR Method Library and the R Package Library. Together, these components provide structured knowledge for MRKB-guided code repair during MR analysis execution.

The MR Method Library provides method-level specifications for automated code repair. As summarized in Table S1, it covers conventional and robust MR methods such as IVW regression (Smith and Ebrahim 2003), MR-Egger regression (Bowden *et al.* 2015), weighted median (Bowden *et al.* 2016), weighted mode (Hartwig *et al.* 2017), MR-PRESSO (Verbanck *et al.* 2018), MRMix (Qi and Chatterjee 2019), GSMR (Zhu *et al.* 2018), CAUSE (Morrison *et al.* 2020), and related methods. Each entry specifies the corresponding package, function, input requirements, key parameters, outputs, and example code.

The R Package Library records package- and function-level knowledge for MR-related R packages, including Mendelian Randomization (Yavorska and Burgess 2017) and TwoSampleMR (Hemani *et al.* 2018). These structured specifications enable targeted repair based on the diagnosed error type, failed method, or failed function.

### 2.7 Validation and repair mechanism

MR-MAS supports two execution modes: automatic execution and human-in-the-loop execution. In the automatic execution mode, the user provides only the initial MR research objective, and the system executes the subtasks according to the predefined stage order. In the human-in-the-loop mode, checkpoints are introduced after key stages, allowing the user to inspect intermediate outputs and decide whether to proceed to the next stage or rerun the current stage.

To describe the execution control between stages, the framework defines a set of stage transition actions: $a_{i}\in \{\text{Proceed},\text{Retry},\text{Repair},\text{Stop}\}$, where $a_{i}$ denotes the control action taken after the completion or abnormal execution of stage $s_{i}$. Proceed indicates that the workflow moves to the next stage; Retry indicates that the current stage is rerun based on user feedback; Repair indicates that error-guided repair is triggered when code execution fails; and Stop indicates that the workflow is terminated when critical inputs are missing or the error cannot be recovered.

During error-guided code repair, runtime errors, failed code, and execution logs are written into short-term memory. The system then retrieves relevant MRKB entries according to the error type or failed function, and injects the retrieved method or tool descriptions into the repair prompt of the current-stage agent, denoted as ${\tilde{p}}_{i}^{\mathrm{repair}}=[p_{i},p_{\mathrm{sys}}^{\mathrm{repair}},K_{i}]$, where $p_{i}$ denotes the original prompt of the stage agent, $p_{\mathrm{sys}}^{\mathrm{repair}}$ denotes the system prompt for error diagnosis and code repair, and $K_{i}$ denotes the relevant method or tool descriptions retrieved from MRKB.

The current-stage agent generates repaired code based on the enhanced repair prompt, failed code, error message, and short-term memory: $c_{i}^{\mathrm{repair}}=A_{i}({\tilde{p}}_{i}^{\mathrm{repair}},c_{i},e_{i},M_{i}^{\mathrm{short}})$, where $A_{i}$ denotes the agent responsible for stage $s_{i}$, $c_{i}$ denotes the failed code, $e_{i}$ denotes the runtime error, $M_{i}^{\mathrm{short}}$ denotes the short-term memory of the current stage, and $c_{i}^{\mathrm{repair}}$ denotes the repaired code. The repaired code is then re-executed. If the repair succeeds, its output is used for the subsequent steps of the current stage; otherwise, the repair process continues until the maximum number of repair rounds is reached, after which the system takes the Stop action and returns the failure reason.

### 2.8 Multi-agent workflow for automated MR research

The end-to-end MR workflow consists of six stages (Fig. 2): (1) research plan formulation, (2) GWAS data acquisition and preprocessing, (3) MR analysis execution, (4) result interpretation, (5) scientific paper generation and (6) paper review. In the following, we describe each stage in detail together with the corresponding division of labour among agents.

**Figure 2:**
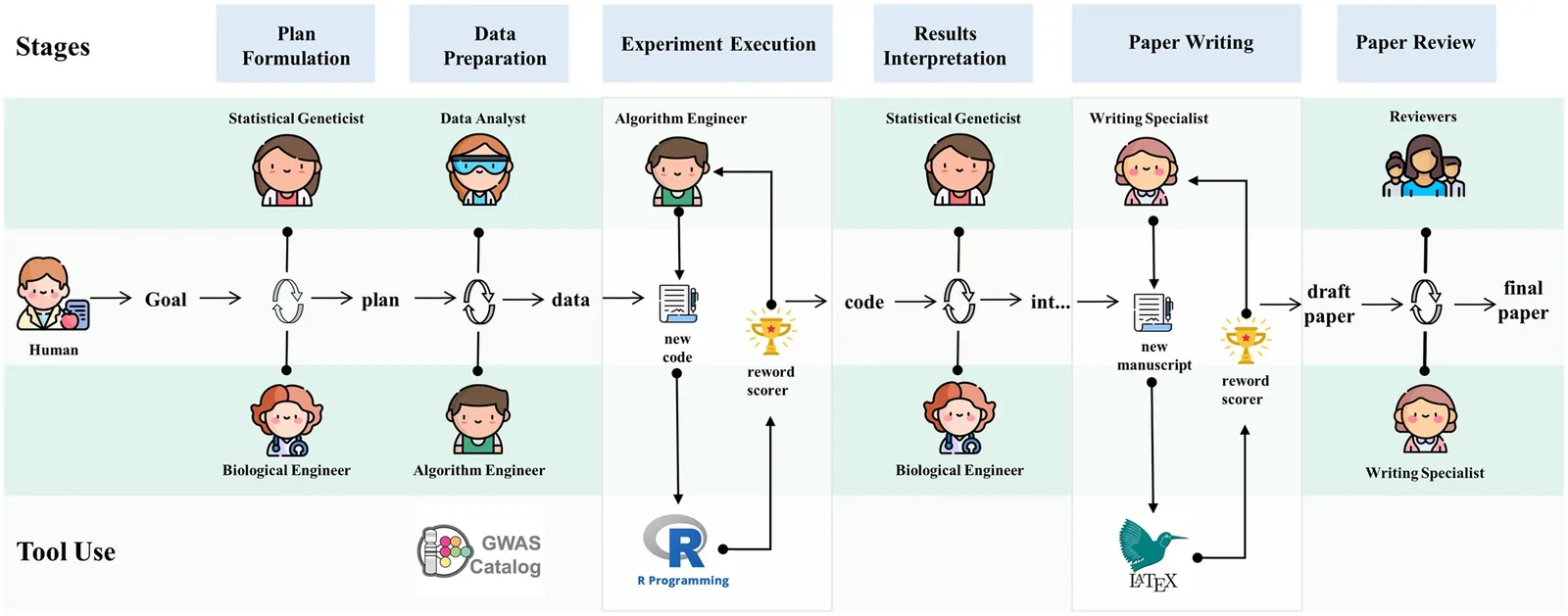
Workflow of MR-MAS for end-to-end automated MR research. The workflow contains six sequential stages from research planning to manuscript review, during which specialized agents exchange structured artefacts and interact with shared resources and external tools to support reproducible analysis.

#### 2.8.1 Stage 1: planning research

In this stage, the biological engineer and statistical geneticist refine the user-defined research objective into an executable analysis plan. Through structured dialogue, they identify the exposure and outcome, design search terms for GWAS data retrieval, define instrumental variable criteria, select MR methods and plan sensitivity analyses. The finalized plan is submitted via a PLAN command and stored as a structured document that serves as the execution blueprint for subsequent stages.

#### 2.8.2 Stage 2: acquiring and preprocessing GWAS data

Guided by the research plan, this stage retrieves GWAS summary statistics from the GWAS Catalog and converts them into standardized input datasets for MR analysis. The data preparation module consists of four core components: TraitMatcher for search-term normalization and trait matching, GWASDataSearch for candidate GWAS study retrieval and quality ranking, GWASLoaderTool for robust data downloading and integrity checking, and standardized preprocessing for instrumental variable selection and allele harmonization.

In this stage, the data analyst agent is responsible for invoking the relevant tools, selecting candidate GWAS datasets, and determining preprocessing strategies, while the algorithm engineer agent generates and optimizes the corresponding preprocessing scripts. The system then automatically validates the executability of these scripts and archives the validated scripts as reusable data preparation programs.

TraitMatcher is designed to reduce heterogeneity in trait names during GWAS retrieval. It first normalizes user-provided terms into standard English expressions aligned with the terminology of the GWAS Catalog. It then combines keyword-based recall with semantic ranking based on TF-IDF similarity (Aizawa 2003) to identify the most relevant trait candidates. The highest-ranked candidate traits are used as standardized inputs for downstream GWAS retrieval. The detailed procedure of TraitMatcher is provided in Supplementary Algorithm S1.

GWASDataSearch ranks candidate GWAS studies using a composite quality score. The scoring factors include sample size, study recency, availability of summary statistics, SNP information, and cohort quality. Then, GWASLoaderTool downloads the selected datasets using automatic retry and integrity-checking mechanisms, thereby reducing the risk that corrupted or incomplete files affect downstream analyses.

During preprocessing, the system first selects SNPs that are associated with the exposure at genome-wide significance as candidate instrumental variables. It then performs LD clumping using the European reference panel from the 1000 Genomes Project Phase 3, with the initial parameters set to r² = 0.001 and a window size of 10 000 kb. To avoid situations in which too few or too many instrumental variables are retained for different exposure–outcome combinations, the system further introduces a dynamic pruning strategy, which adaptively adjusts LD parameters according to the number of SNPs retained after clumping. The system then removes weak instruments based on the F-statistic and harmonizes effect alleles between the exposure and outcome datasets before MR analysis.

#### 2.8.3 Stage 3: executing MR analysis

The MR analysis execution stage is organized as a closed-loop process consisting of code generation, execution, error diagnosis, MRKB-guided repair, scoring, and optimization. The formal description of this process is provided in Supplementary Algorithm S2. The algorithm engineer agent generates an executable R analysis script based on the research plan and the prepared GWAS datasets. The system then automatically runs the script to detect syntax errors or runtime errors.

If script execution fails, the workflow invokes the error-guided repair mechanism described in Section 2.7. Specifically, the failed code, execution log, and error message are written into short-term memory and used to retrieve relevant MRKB entries, which guide the algorithm engineer agent in performing targeted code repair. The repaired script is then re-executed until it runs successfully or the maximum number of repair rounds is reached.

After successful execution, the system uses a prompt-based LLM scorer (Supplementary Note S1) as an internal quality-control mechanism to evaluate whether the generated MR analysis script is complete, consistent with the research plan, and reproducible. Scripts with scores below the predefined threshold are further optimized; otherwise, the script is accepted and archived. The finalized script performs causal effect estimation, sensitivity analyses, and result visualization, and stores the estimates, figures, and key intermediate artefacts for downstream result interpretation.

#### 2.8.4 Stage 4: interpreting results

The goal of the result interpretation stage is to distill meaningful scientific insights from the outputs of the MR analyses and provide a basis for the final manuscript. In this stage, the statistical geneticist and biological engineer agents work collaboratively to systematically evaluate the causal effect estimates, significance test results, sensitivity analysis outputs, and the consistency across multiple MR methods. Once they reach a consensus on the interpretation, the statistical geneticist agent submits it via an INTERPRETATION command, which then serves as the foundation for the subsequent manuscript drafting stage.

#### 2.8.5 Stage 5: generating scientific paper

In this stage, the writing specialist agent transforms the analysis outputs into a structured scientific manuscript draft. It first constructs a LaTeX framework following the standard structure of scientific articles, including the abstract, introduction, methods, results, and discussion sections. In this study, the generated files are configured according to the OUP General Template (OUP General Template available at https://www.overleaf.com/latex/templates/oup-general-template/ybpypwncdxyb.). The system also retrieves relevant literature from PubMed and PubMed Central to support the writing of the research background, related work, and result interpretation sections.

Subsequently, the writing specialist agent generates and refines the manuscript section by section based on the paper framework, analysis results, and literature context. LaTeX compilation checks are performed after each update to ensure structural and formatting consistency. All automatically generated text is treated as a draft and requires further inspection, editing, and final confirmation by human researchers. Therefore, the system is positioned as an assistant for the MR research workflow rather than a replacement for domain experts.

#### 2.8.6 Stage 6: reviewing paper

In the paper reviewing stage, multiple reviewer agents first generate independent assessments, simulating journal peer review by scoring and commenting on the draft along dimensions such as novelty, methodological rigor, clarity of presentation, and scientific significance. The writing specialist agent then integrates these evaluations to determine whether the manuscript can be considered complete and the workflow terminated, or whether it should return to earlier stages such as planning, experimentation, result interpretation, or writing for targeted revisions.

## 3 Results

We evaluate MR-MAS in terms of analytical consistency, trait retrieval robustness, workflow efficiency, and reproducibility. For analytical consistency, MR-MAS was run with gpt-4o (Hurst *et al.* 2024) on exposure–outcome pairs spanning five prior-evidence categories. TraitMatcher is evaluated using Top-*k* hit rates under noisy query settings, with the reported results averaged across all four base model backends: o1 (Jaech *et al.* 2024), gpt-4o, o1-mini (Jaech *et al.* 2024), and DeepSeek-V3.2-Exp (DeepSeek-AI 2025). Detailed model-backend information is provided in Table S2. Workflow efficiency and operational stability are measured across the same four backends using runtime, API cost, and stage-level success rate. Reproducibility is examined under the same four backends by repeating the complete workflow 10 times for each backend under identical prompts and comparing both causal estimates and generated analysis scripts. The GWAS datasets used in the main evaluation are summarized in Table S3.

### 3.1 MR-MAS reproduces evidence-supported MR signals

To evaluate whether MR-MAS can generate scientifically plausible causal interpretations on real GWAS research questions, we assessed its performance using exposure–outcome pairs stratified into five prior-evidence categories (Morrison *et al.* 2020): considered causal, supported by literature, unknown or conflicting evidence, implausible or unsupported, and considered non-causal. These categories are described in Supplementary Note S2. For each pair, we applied five core MR methods: IVW, MR-Egger regression, weighted median, weighted mode, and MR-PRESSO, and recorded the corresponding *P*-values and effect directions.

The results are summarized in Table 1. In the considered causal group, most trait pairs showed nominally significant estimates in the expected direction across multiple MR methods. Results were more heterogeneous in the literature-supported and unknown or conflicting groups, suggesting greater sensitivity to estimator choice and available instruments. No consistent nominally significant signals were observed in the implausible or unsupported and considered non-causal groups. Instrument strength and statistical power for each pair are summarized in Table S4. Mean F-statistics ranged from 35.71 to 83.91, with all retained instruments satisfying the prespecified F>10 criterion. Estimated power varied across pairs, warranting cautious interpretation of some null findings.

**Table 1:** Method-level MR results across GWAS trait pairs stratified by prior biomedical and MR evidence.

Trait pair	IVW	MR-Egger	Wtd Median	Wtd Mode	MR-PRESSO
Considered causal					
SBP → CAD	**3.15** × **10**${\;}^{-6}$ ↑	**0.0014** ↑	**3.32** × **10**${\;}^{-8}$ ↑	**0.00035** ↑	**7.97** × **10**${\;}^{-9}$ ↑
DBP → CAD	**0.0028** ↑	0.17 ↑	**1.92** × **10**${\;}^{-5}$ ↑	**8.97** × **10**${\;}^{-5}$ ↑	**1.06** × **10**${\;}^{-5}$ ↑
SBP → Stroke	**0.0028** ↑	**0.010** ↑	**0.0065** ↑	0.05 ↑	**0.0045** ↑
DBP → Stroke	**0.0019** ↑	0.20 ↑	**0.018** ↑	0.06 ↑	**3.82** × **10**${\;}^{-5}$ ↑
LDL → CAD	**4.59** × **10**${\;}^{-5}$ ↑	**0.0062** ↑	**0.00070** ↑	0.29 ↑	**6.03** × **10**${\;}^{-5}$ ↑
LDL → Stroke	0.8324 ↑	0.0507 ↑	0.4376 ↑	0.2278 ↑	0.5373 ↑
Supported by literature					
BMI → CAD	**0.0011** ↑	0.099 ↑	**5.70** × **10**${\;}^{-5}$ ↑	**0.022** ↑	**5.86** × **10**${\;}^{-6}$ ↑
BMI → T2D	0.068 ↑	0.32 ↑	**6.61** × **10**${\;}^{-11}$ ↑	**0.0047** ↑	**1.66** × **10**${\;}^{-6}$ ↑
BMI → Stroke	0.5847 ↑	0.1208 ↑	0.5530 ↑	0.8346 ↑	0.8291 ↑
BFP → CAD	**0.0062** ↑	0.26 ↑	**2.62** × **10**${\;}^{-6}$ ↑	**8.62** × **10**${\;}^{-5}$ ↑	**1.00** × **10**${\;}^{-6}$ ↑
BFP → T2D	**0.00041** ↑	0.71 ↓	**8.90** × **10**${\;}^{-6}$ ↑	**0.0097** ↑	**9.53** × **10**${\;}^{-8}$ ↑
BFP → Stroke	0.2327 ↑	0.8500 ↓	0.0803 ↑	0.2276 ↑	0.2399 ↑
Unknown or conflicting evidence					
SBP → T2D	**0.0198** ↑	0.8188 ↓	0.1233 ↑	0.1336 ↑	0.1051 ↑
BW → CAD	0.5615 ↑	0.8219 ↑	0.4071 ↓	0.1882 ↓	0.2579 ↑
BMI → Asthma	**5.49** × **10**${\;}^{-5}$ ↑	0.3821 ↑	**0.0111** ↑	0.2501 ↑	**0.00035** ↑
BW → Stroke	0.1553 ↑	0.2745 ↑	0.0807 ↑	0.2411 ↓	0.1644 ↑
BFP → Asthma	**1.09** × **10**${\;}^{-5}$ ↑	0.7602 ↓	**0.00083** ↑	**0.0318** ↑	**2.22** × **10**${\;}^{-5}$ ↑
BW → T2D	**0.0275** ↑	0.8040 ↑	**0.0428** ↑	0.9111 ↓	**0.0051** ↑
Implausible or unsupported					
SBP → Asthma	0.8316 ↑	0.7621 ↑	0.3548 ↓	0.5454 ↓	0.8370 ↑
DBP → Asthma	0.7778 ↓	0.3563 ↓	0.2434 ↓	0.1929 ↓	0.4612 ↓
HDL → Asthma	0.4081 ↓	0.5130 ↓	0.7828 ↓	0.4575 ↑	0.3439 ↓
LDL → Asthma	0.3666 ↓	0.5562 ↓	0.3582 ↓	0.1947 ↓	0.2640 ↓
Alcohol → Asthma	0.9111 ↓	0.2794 ↑	0.6652 ↑	0.8355 ↑	0.9131 ↓
Height → T2D	0.9529 ↑	0.2368 ↑	0.4767 ↑	0.4273 ↑	0.9531 ↑
Considered non-causal					
HDL → CAD	0.6178 ↑	0.9738 ↓	0.4932 ↑	0.7610 ↑	0.5089 ↑
HDL → Stroke	0.9246 ↑	0.6536 ↓	0.8469 ↑	0.8673 ↑	0.9250 ↑
CRP → IS	0.2506 ↓	0.7562 ↓	0.6393 ↓	0.5367 ↓	0.4814 ↓
Adiponectin → T2D	0.9277 ↓	0.7585 ↑	0.7272 ↓	0.6025 ↓	0.9445 ↓

*Note:* Columns 2–6 give the *P* value for each MR method. Values are in bold if p<0.05. Arrows indicate the sign of the corresponding effect estimate.

Abbreviations: BFP, body fat percentage; BMI, body mass index; BW, body weight; CAD, coronary artery disease; DBP, diastolic blood pressure; HDL, high density lipoprotein; LDL, low density lipoprotein; SBP, systolic blood pressure; T2D, type 2 diabetes; CRP, C-reactive protein; IS, ischemic stroke.

We further compared MR-MAS with MRAgent on the same 10 evaluation pairs from the implausible or unsupported and considered non-causal groups under their respective default workflows. MRAgent reported three definitive positive causal associations, whereas MR-MAS reported none. To investigate the source of these discrepancies, we conducted a harmonized-input analysis by rerunning the three positive cases from MRAgent using the identical harmonized datasets within MR-MAS. Under identical inputs, MR-MAS reproduced two of the three positive associations while maintaining a null conclusion for the remaining case. These findings indicate that differences in upstream data processing contribute to the discrepancies observed under the default workflows but do not fully explain them, highlighting that both upstream processing and the interpretation strategy influence the final causal assessment. Further details are provided in Supplementary Note S3.

Overall, MR-MAS reproduced several literature-supported MR signals, while no consistent nominally significant signals were observed for the evaluated implausible, unsupported, or considered non-causal pairs under its default workflow.

### 3.2 TraitMatcher is robust to noisy inputs

To evaluate the robustness of TraitMatcher in standardizing trait expressions and supporting GWAS retrieval, we tested whether it could identify target traits from curated candidate sets containing semantically similar entries under realistic noisy retrieval settings.

The benchmark includes 24 target traits, each paired with a unique GWAS Catalog entry as the gold standard, with seven query variants per trait covering five expression types, yielding 168 queries in total.

For each query, TraitMatcher generated a ranked candidate list using TF–IDF cosine similarity followed by the re-ranking strategy described above. Retrieval performance was evaluated using Hit@*k* (k=1,2,3), with Precision@*k* and F1@*k* included as supplementary metrics. A literal substring-matching method was used as a non-semantic baseline. Detailed metric definitions and baseline implementation are provided in Supplementary Note S4.

After computing these metrics, we aggregated the results across all 24 traits and further performed stratified analyses by query type. As shown in Fig. 3, TraitMatcher consistently outperformed the baseline. Averaged across base models, the hit rates reached 0.89 for Top-1, 0.90 for Top-2, and 0.91 for Top-3, representing an improvement of about 76%–77% over the baseline. The small gap between Top-1 and Top-3 suggests that correct matches were usually ranked near the top.

**Figure 3:**
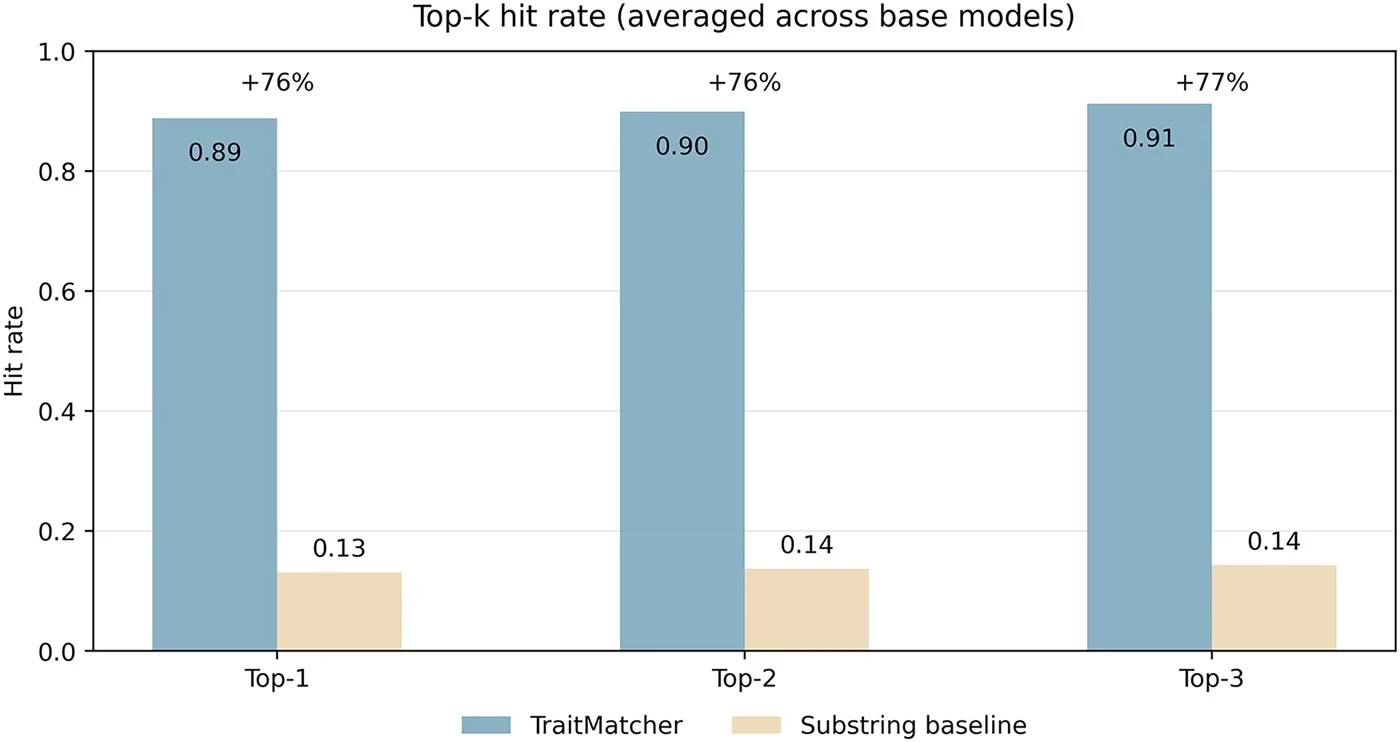
Average Top-*k* hit rate of TraitMatcher compared with the substring matching baseline. Bars show the mean Top-1, Top-2, and Top-3 hit rates across all base models, with labels indicating absolute values and relative improvements.

Figure 4 reports performance stratified by query type. TraitMatcher performs well across categories, with Top-1 hit rates above 0.85 for all types. Chinese and misspelling queries achieved the highest scores, while synonym queries were slightly lower. Increasing *k* from 1 to 3 consistently improved performance, indicating that correct matches were typically captured within the top three candidates.

**Figure 4:**
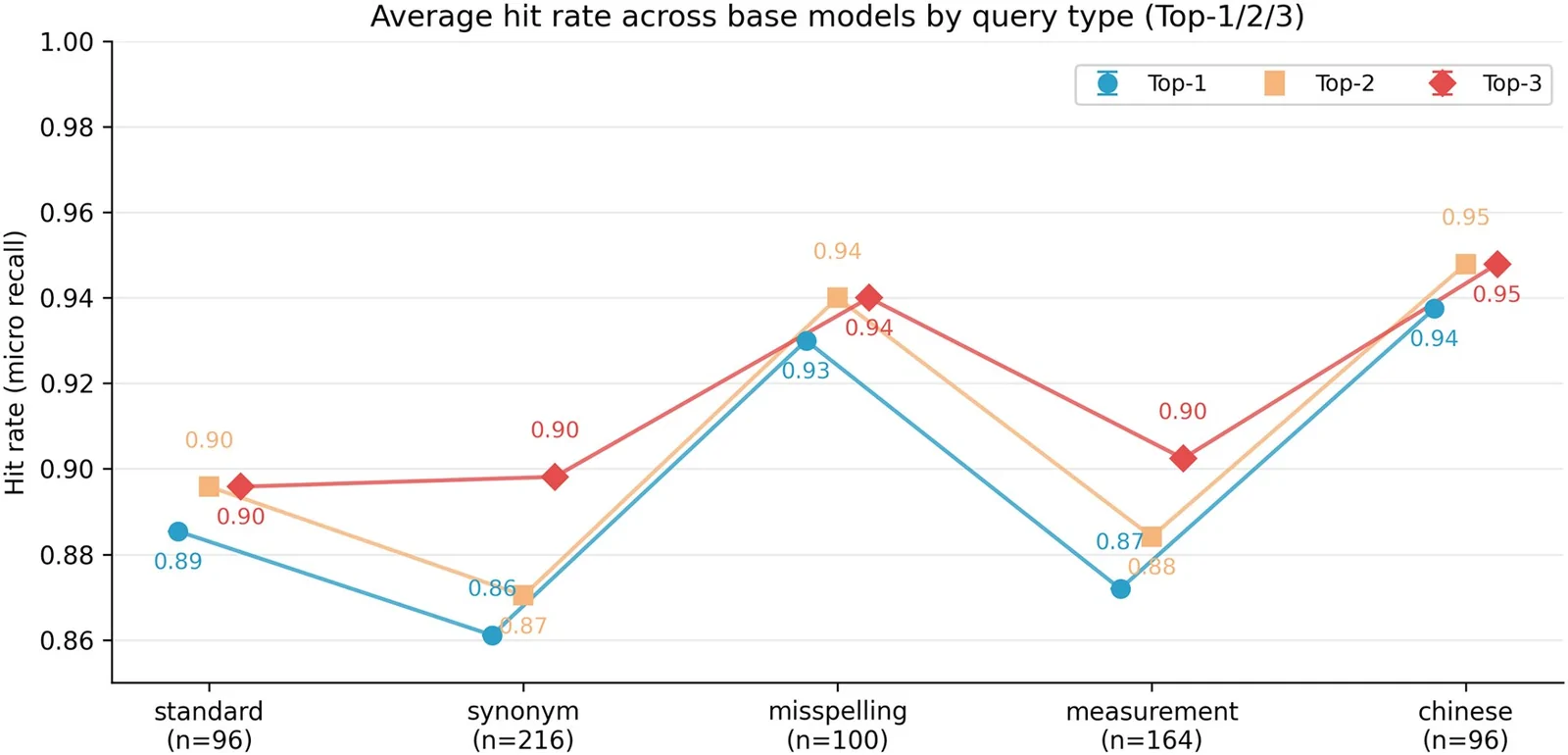
Average hit rate of TraitMatcher across different query types. Each point shows the mean performance over all four base models.

Table 2 shows that o1-mini achieved the highest overall F1 score, followed by o1, while gpt-4o and deepseek-V3.2-Exp performed slightly worse, suggesting differences in their ability to prioritize semantically relevant traits.

**Table 2:** Comparison of precision, recall and F1 at Top-*k* across different base models under the micro definition.

Model	k=1			k=2			k=3			Avg F1
	Precision	Recall	F1	Precision	Recall	F1	Precision	Recall	F1	
o1	0.9524	0.9524	0.9524	0.4792	0.9583	0.6389	0.3194	0.9583	0.4792	0.6902
gpt-4o	0.7976	0.7976	0.7976	0.4167	0.8333	0.5556	0.2837	0.8512	0.4256	0.5929
o1-mini	**0.9643**	**0.9643**	**0.9643**	**0.4821**	**0.9643**	**0.6429**	**0.3274**	**0.9821**	**0.4911**	**0.7028**
deepseek-V3.2-Exp	0.8214	0.8214	0.8214	0.4107	0.8214	0.5476	0.2817	0.8452	0.4226	0.5972

*Note:* Metrics are computed under the micro definition. Each query contributes at most one true positive within Top-*k*, hence recall equals the Top-*k* hit rate. Bold values denote the best in each column.

### 3.3 Trade-off of runtime, cost, and success rate among different base models

To evaluate the computational efficiency, cost, and operational reliability of MR-MAS, we measured runtime, API cost, and success rate across the six workflow stages. Figure S1 and Table 3 summarize the stage-wise and overall runtime and cost results. MR Analysis had the highest average runtime, whereas Paper Writing had the highest average cost.

**Table 3:** Average runtime and cost of each subtask.

Model	**Plan Formulation**		**Data Preparation**		**MR Analysis**		**Results Interpretation**		**Paper Writing**		**Paper Review**		**Entire Workflow**	
	T	C	T	C	T	C	T	C	T	C	T	C	T	C
o1	29.23s	$0.13	**184.97s**	$0.55	**265.83s**	$0.68	35.30s	$0.13	815.57s	$5.60	148.12s	$0.46	**1479.02s**	$7.55
gpt-4o	97.28s	$0.06	468.78s	$0.12	2253.01s	$0.67	**30.06s**	$**0.06**	763.75 s	$2.13	**34.24s**	$0.05	3647.12s	$3.09
o1-mini	**19.42s**	$**0.02**	605.06 s	$0.17	3573.44s	$1.87	65.48s	$0.13	**435.72s**	$0.97	42.18s	$0.05	4741.30s	$3.21
DS-V3.2-Exp	100.62s	$**0.02**	222.98 s	$**0.04**	421.20 s	$**0.05**	166.49 s	$0.10	858.79s	$**0.29**	139.51 s	$**0.03**	1909.59s	$**0.53**
Avg	61.63s	$0.06	370.45s	$0.22	1628.37s	$0.82	74.33s	$0.11	718.46s	$2.25	91.01s	$0.15	2944.26s	$3.60

*Note:* T and C denote runtime in seconds and API cost in USD, respectively. DS-V3.2-Exp abbreviates deepseek-V3.2-Exp.

In terms of overall runtime, o1 was the fastest (1479.02 s), followed by deepseek-V3.2-Exp (1909.59s), gpt-4o (3647.12 s), and o1-mini (4741.30 s). At the stage level, o1 was faster in Data Preparation and MR Analysis, whereas gpt-4o was faster in Results Interpretation and Paper Review. In terms of cost, deepseek-V3.2-Exp had the lowest total cost ($0.53), whereas o1 had the highest ($7.55); gpt-4o and o1-mini were intermediate at approximately $3.

Table 4 summarizes the stage-level success rates. All models achieved 100% success in Plan Formulation, Data Preparation, Results Interpretation, Paper Writing, and Paper Review, with failures confined to MR Analysis. o1 showed the highest stability, followed by o1-mini, whereas gpt-4o and deepseek-V3.2-Exp had lower success rates in this stage. To further assess repair robustness, we compared MRKB-guided repair with plain LLM repair in Supplementary Note S5. MRKB-guided repair achieved higher repair success (60/60 vs. 34/60 tasks) and required fewer mean repair rounds (4.98 vs. 8.63).

**Table 4:** Success rate of each subtask.

Model	Plan formulation	Data preparation	MR analysis	Results interpretation	Paper writing	Paper review	Mean stage success
o1	100%	100%	100%	100%	100%	100%	**100%**
gpt-4o	100%	100%	70%	100%	100%	100%	95%
o1-mini	100%	100%	90%	100%	100%	100%	98.3%
deepseek-V3.2-Exp	100%	100%	70%	100%	100%	100%	95%

*Note:* Success rate is defined as the proportion of runs in which each subtask is completed without failure.

Overall, the four backends exhibited distinct trade-offs among runtime, cost, and operational stability, with o1 favouring runtime and stability and deepseek-V3.2-Exp favouring cost.

### 3.4 MR-MAS demonstrates robust performance across multiple runs

To assess the stability and reproducibility of the proposed automated MR workflow under stochastic code generation by LLMs, we repeated the entire workflow 10 times for each model backend using identical prompts. We then compared both the resulting causal effect estimates and the generated analysis scripts across runs.

As shown in Fig. 5, the causal inference results remained highly consistent across repeated executions. Estimated effect sizes clustered tightly across runs and model backends, and the statistical significance patterns were unchanged, indicating that stochastic variations in code generation did not materially affect the final causal inference results.

**Figure 5:**
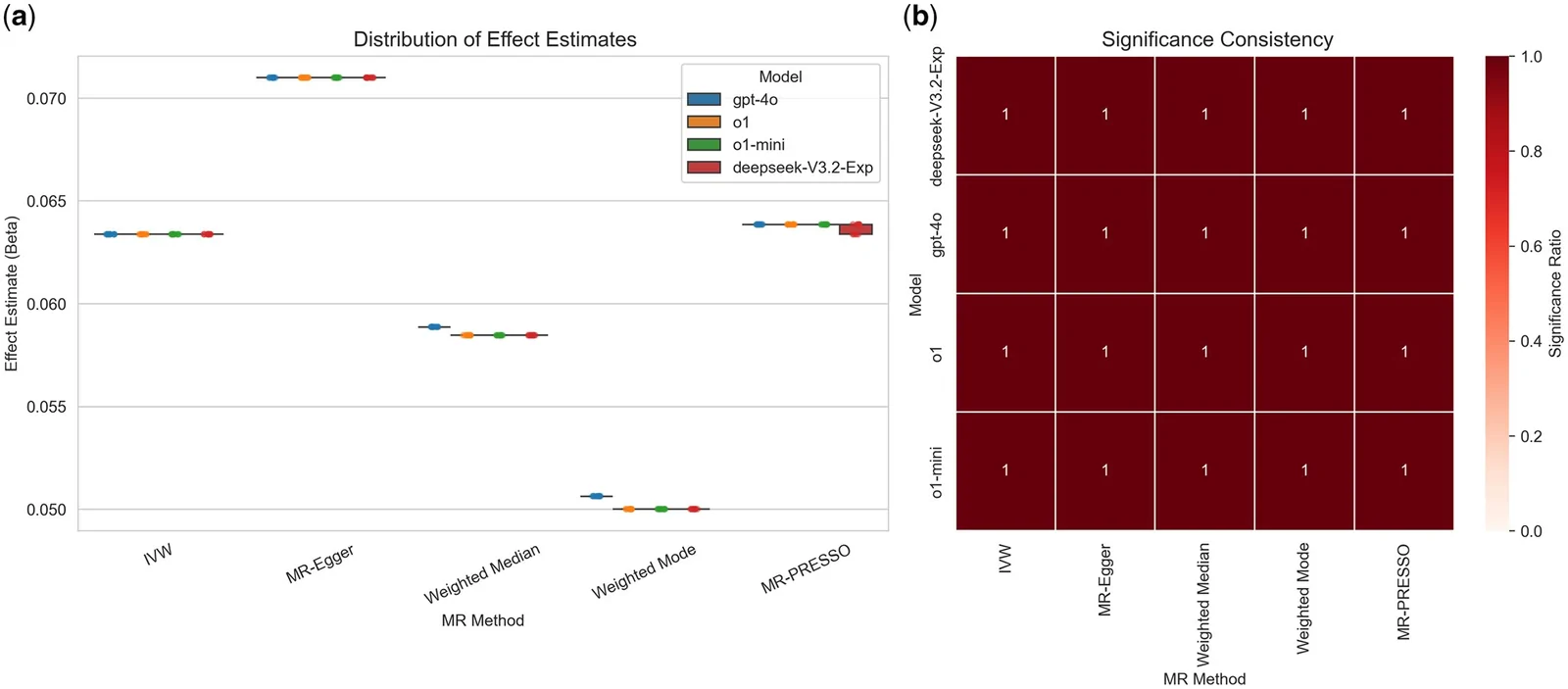
Stability of causal effect estimates across repeated workflow executions. (a) Distribution of causal effect estimates obtained from 10 repeated runs of the automated MR workflow under identical inputs for four model backends. Each point represents the estimated effect size from one run. (b) Consistency of statistical significance across repeated runs for different MR methods. Each cell indicates the proportion of runs in which the corresponding method produced a statistically significant result.

As shown in Fig. 6, the generated analysis scripts exhibited moderate textual variability across runs. Pairwise script similarity was quantified using Python’s SequenceMatcher (SequenceMatcher is provided by Python’s difflib module; see https://docs.python.org/3/library/difflib.html.), yielding values ranging from approximately 0.14 to 0.40 across model backends. These differences mainly reflected variations in variable naming, logging statements, function-call styles, and other minor implementation details. Despite such syntactic variation, all scripts consistently implemented the same MR analysis procedure and produced highly consistent causal estimates.

**Figure 6:**
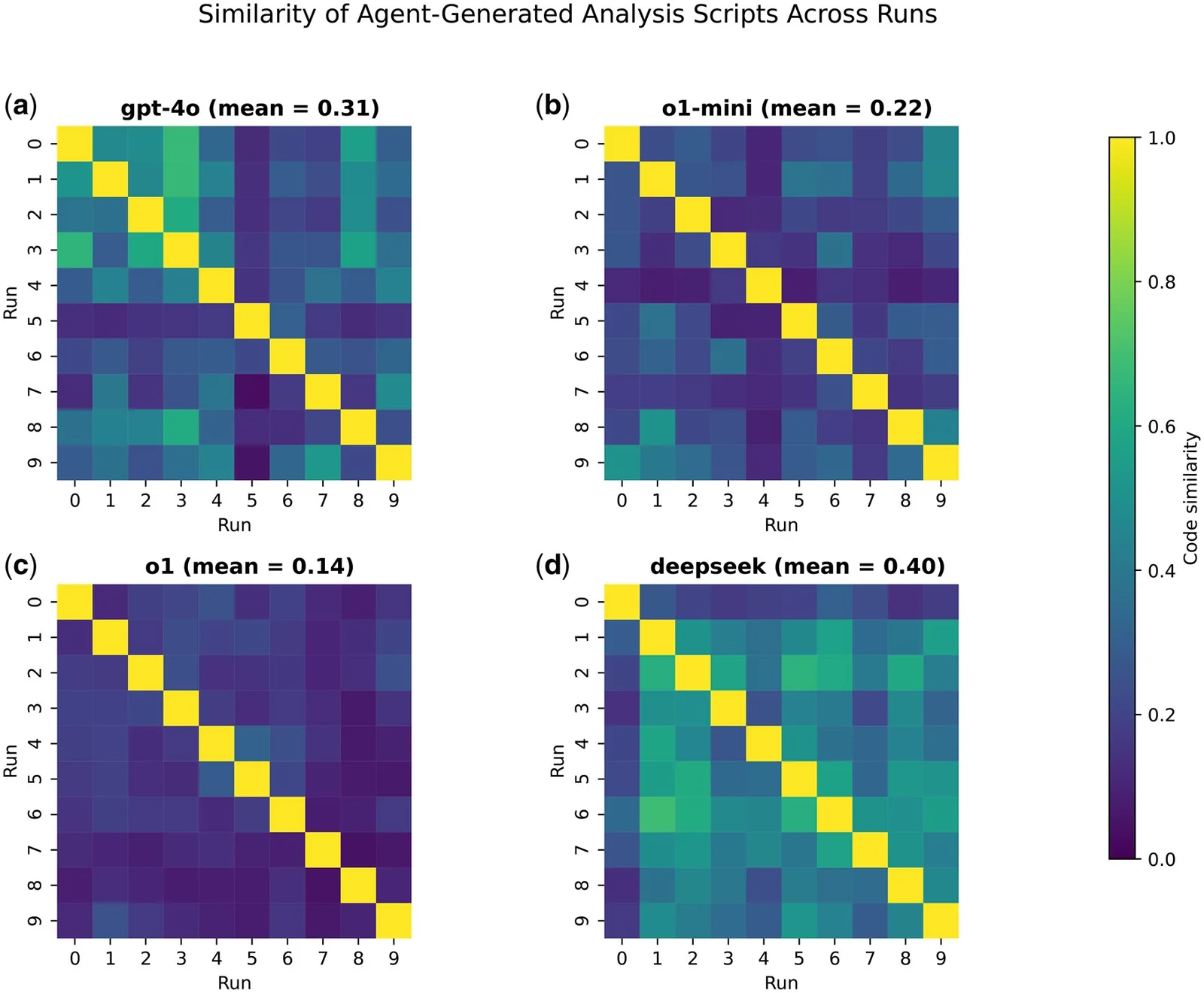
Similarity of analysis scripts across repeated workflow executions. Pairwise similarity was calculated using the SequenceMatcher algorithm for scripts generated under identical prompts and input data. Code-generation calls used a fixed temperature of 1.0 for each model backend. Panels (a)–(d) show the pairwise code similarity for gpt-4o, o1-mini, o1, and deepseek (short for deepseek-V3.2-Exp), respectively. The bright yellow diagonal represents perfect self-similarity (1.0), while the off-diagonal cells indicate similarity between scripts generated in different runs.

In a separate system-level comparison, we further evaluated MR-MAS against Biomni and Claude Code (https://www.claude.com/product/claude-code) using MR workflow tasks. Under the evaluated settings, MR-MAS showed higher workflow reproducibility in both comparisons. Further details of the experimental setup and results are provided in Supplementary Note S6.

Overall, these results indicate that MR-MAS maintained consistent workflow execution across repeated runs despite stochastic variation in LLM-generated code.

## 4 Discussion

This study proposes MR-MAS, a multi-agent automated MR system that reduces the complexity and manual burden of traditional MR workflows, enabling largely automated MR analysis. By integrating study planning, GWAS data preparation, MR analysis, result interpretation, and manuscript generation into a unified pipeline, MR-MAS is designed to lower the technical barrier to standardized MR analysis while still requiring expert review for publication-level interpretation.

The experimental results characterize the performance of MR-MAS from several perspectives. In the analytical evaluation, several pairs with prior literature support showed nominally significant and directionally consistent estimates, whereas no consistent nominally significant signals were observed for the evaluated implausible, unsupported, or considered non-causal pairs. At the retrieval level, TraitMatcher maintains high accuracy under noisy and multilingual queries, supporting robust phenotype identification in realistic research scenarios. At the system level, MR-MAS also exhibits clear trade-offs among runtime, cost, and success rate across different model backends, providing flexibility for different usage priorities. Furthermore, repeated-run experiments show that although LLM-driven code generation introduces moderate textual variability in the generated scripts, the causal estimates remain highly consistent across runs. Together, these findings suggest that MR-MAS may reduce manual workload while maintaining a useful balance among analytical consistency, operational efficiency, and workflow reproducibility.

Several limitations remain. The current workflow still relies on a largely predefined stage structure and lacks dynamic scheduling based on runtime conditions. Although the final causal estimates were generally stable, stochasticity in LLM outputs may still affect intermediate decision making. In addition, MR-MAS cannot eliminate MR-specific biases, and its causal interpretations remain conditional on data quality and MR assumptions. The current study also did not include a formal user study involving non-specialist users. Therefore, MR-MAS outputs should still be reviewed by domain experts before being used as publication-level scientific evidence. Future work will focus on introducing dynamic planning and scheduling mechanisms, strengthening randomness control and validation, and improving system robustness for large-scale causal research.

## 5 Conclusion

In conclusion, we propose and evaluate MR-MAS, a multi-agent automated MR system that integrates fragmented analytical steps into a standardized end-to-end workflow. The results suggest that MR-MAS can support methodologically consistent MR analysis, robust trait retrieval, and stable workflow execution across the evaluated settings. MR-MAS may provide practical support for large-scale, standardized, and reproducible automated MR studies and future methodological extensions.

## Supplementary Material

btag722_Supplementary_Data

## Data Availability

The GWAS summary statistics used in this study were obtained from previously published studies, with their sources and corresponding PubMed identifiers (PMIDs) provided in the Supplementary Materials. The source code, MRKB, and benchmark data are publicly available at https://github.com/Zaiwen/MR-MAS. The version used in this study has been archived at https://doi.org/10.5281/zenodo.20342636.
