# Supplementary material for: MR-MAS: an end-to-end multi-agent system for autonomous Mendelian randomization analysis
Source: Bioinformatics. 2026 Oct 20;42(10):btag722. doi: 10.1093/bioinformatics/btag722 (PMC13680005; doi:10.1093/bioinformatics/btag722)
Supplement: Supplementary file 1 [file btag722_supplementary_data.pdf]

Supplementary Materials

Supplementary Note S1. Prompt-based LLM scorer for MR code quality control.

In Stage 3, after successful code execution, the reward scoring module was used to evaluate whether the generated MR analysis code and its corresponding execution output were sufficiently complete and consistent with the predefined research plan. This module was implemented as a prompt-based LLM scorer and served primarily as an internal quality-control mechanism for workflow management, rather than as an independent measure of scientific validity.

The scorer was implemented using a fixed structured prompt. This prompt instructed the LLM to act as a bioinformatics expert and to evaluate the generated MR analysis based on three inputs: the predefined research plan, the generated MR analysis code, and the corresponding execution output. The scoring criteria included the completeness and correctness of the MR analysis pipeline, the consistency between the generated code and the research plan, the interpretability of the MR results, the clarity and reproducibility of the code, and robustness to common execution or data-processing issues. The complete prompt template used for the prompt-based LLM scorer is provided in Box S7c.

The scorer returns a normalized score ranging from 0 to 1, together with a brief justification. Higher scores indicate that the generated script is more complete, more consistent with the research plan, more reproducible, and capable of producing interpretable MR results. Lower scores indicate incomplete implementation, deviation from the planned procedure, insufficient result reporting, or unresolved execution and data-processing issues.

The default acceptance threshold was set to 0.80. Outputs with scores below 0.80 were returned for further refinement, whereas outputs with scores at or above 0.80 were accepted as finalized scripts and archived in the results repository. This threshold was empirically selected as a practical workflow-control cutoff to balance script quality control against the additional cost of repeated refinement.

It is important to note that this threshold was used only to determine whether the generated code should undergo further refinement. It did not directly determine the final causal conclusion. The final causal conclusions were still derived from MR effect estimates, sensitivity analysis results, and evidence-based interpretation criteria. Therefore, changing this threshold may affect the number of refinement rounds, computational cost, and the point at which a script is accepted, but it does not directly define the final causal interpretation.

Table S1: MR methods included in the MR Method Library.

**Method**	**Methodological role**
Inverse variance weighted (IVW)	Conventional MR effect estimation
MR-Egger	Pleiotropy-robust effect estimation
Weighted median	Robust estimation with invalid instruments
Weighted mode	Robust estimation based on modal clustering
MR-PRESSO	Pleiotropy and outlier detection
MRMix	Mixture-model-based robust estimation
GSMR	Summary-data MR with outlier filtering
Contamination mixture [1]	Robust estimation under invalid instruments
MR-GRIP [2]	Genotype recoding-invariant MR estimation
MR-RAPS [3]	Robust estimation with many weak instruments
CAUSE	Genome-wide MR with correlated pleiotropy modeling

Note: This table lists the MR methods currently included in the MR Method Library. The methodological roles are broad descriptions of the main use of each method in MR analysis, rather than mutually exclusive categories.

Supplementary Note S2. Prior causal evidence used for GWAS trait-pair classification

To facilitate an evidence-stratified assessment of Mendelian randomization (MR) conclusions, the genome-wide association study (GWAS) trait pairs presented in the main manuscript Table 1 were categorized based on existing causal evidence. This classification was intended to evaluate whether the automated analytical workflow could reproduce biologically credible MR findings across relationships with different degrees of prior support.

Following the literature-curated evidence categories described in the supplementary materials of Morrison et al. [4], exposure–outcome pairs were assigned to one of five evidence tiers: (1) considered causal

(C), (2) supported by literature (S), (3) unknown or conflicting evidence (U), (4) implausible or unsupported (I), and (5) considered non-causal (N). Within this framework, pairs classified as considered causal have strong and widely accepted support from randomized trials, previous MR studies, or well-established biological mechanisms. Pairs classified as supported by literature have observational and biological support, although the causal evidence is not definitive. Unknown or conflicting evidence indicates that the existing literature is limited, inconsistent, or inconclusive. Implausible or unsupported pairs lack a clear biological rationale or consistent empirical support. Considered non-causal pairs are those for which prior evidence does not support a direct causal effect despite possible observational associations.

Table S2: Model backends used in the system-level evaluation.

**Model backend**	**Provider**	**Model type**	**Public parameter size**
o1	OpenAI	Proprietary reasoning model	Not publicly disclosed
gpt-4o	OpenAI	Proprietary multimodal model	Not publicly disclosed
o1-mini	OpenAI	Proprietary compact reasoning model	Not publicly disclosed
DeepSeek-V3.2-Exp	DeepSeek-AI	Open-weight sparse-attention model	685B parameters

Note: Parameter sizes are reported only when they are publicly available from official model documentation or model cards. For proprietary OpenAI models, the exact parameter sizes are not publicly disclosed; therefore, they are reported as "Not publicly disclosed" rather than using unverifiable third-party estimates.

Table S3: Genome-wide association studies for common diseases and risk factors.

**Abbrev.**	**Trait**	**Sample size**	**Cases**	**Controls**	**PMID**	**First author (Year)**
DBP	Diastolic blood pressure	1028980	–	–	38689001	Keaton (2024)
LDL	Low-density lipoprotein	1020016	–	–	34887591	Graham (2021)
SBP	Systolic blood pressure	1028980	–	–	38689001	Keaton (2024)
HDL	High-density lipoprotein	394642	–	–	39789286	Loya (2025)
BMI	Body mass index	161564	–	–	38987242	Venkatesh (2024)
BFP	Body fat percentage	401772	–	–	34017140	Mbatchou (2021)
BW	Body weight	394642	–	–	39789286	Loya (2025)
Alcohol	Alcohol frequency weekly	283162	–	–	37106081	Schoeler (2023)
Height	Height	458235	–	–	34226706	Barton (2021)
CRP	C-reactive protein	575531	–	–	35459240	Said (2022)
Adiponectin	Adiponectin	47745	–	–	39789286	Loya (2025)
CAD	Coronary artery disease	1378170	210842	1167328	36474045	Aragam (2022)
Asthma	Asthma	1376071	121940	1254131	36777996	Zhou (2022)
T2D	Type 2 diabetes	490089	38841	451248	34594039	Sakaue (2021)
Stroke	Stroke	446696	40585	406111	39537608	Koskeridis (2024)
IS	Ischemic stroke	1296908	62100	1234808	36180795	Mishra (2022)

Note: Sample sizes and case–control counts are reported as in the original GWAS publications.

For trait pairs already included in the evidence-stratified reference set reported by Morrison et al. [4], we retained the corresponding prior-evidence categories. For exposure–outcome pairs that were not covered by that reference set but were additionally included in our evaluation, including those in the exploratory phenotype-diversity analysis, we assigned prior-evidence categories through a separate literature review. In this review, we prioritized pair-specific MR or genetic evidence over observational associations alone. If multiple MR studies were inconsistent, or if the conclusion depended on specific model assumptions or sensitivity analyses, the pair was assigned to the unknown or conflicting evidence category. If observational associations were reported but genetic or MR evidence did not support a direct causal role, the pair was classified as considered non-causal. If direct pair-specific causal evidence was limited but there was consistent biological, phenotypic, or genetic support for a relationship between the traits, the pair was classified as supported by literature rather than considered causal.

These prior-evidence categories were used as literature-informed external references for interpreting the system outputs, rather than as definitive ground-truth labels or post hoc classifications based on the results generated by the proposed workflow.

SI → ALS. This pair was classified as *unknown or conflicting evidence*. Smoking has long been investigated as a possible risk factor for amyotrophic lateral sclerosis (ALS), but the causal evidence

Table S4: Number of instruments, mean F-statistic, and statistical power for each exposure–outcome pair.

**Exposure–outcome pairs**	**nSNP**	**Mean F-statistic**	**Power (%)**
SBP → CAD	49	62.71	99.4
DBP → CAD	44	58.94	98.5
SBP → Stroke	49	62.71	54.2
DBP → Stroke	46	56.13	49.0
LDL → CAD	37	71.11	99.2
LDL → Stroke	37	73.70	52.0
BMI → CAD	28	35.71	99.0
BMI → T2D	28	35.71	86.0
BMI → Stroke	26	35.85	84.8
BFP → CAD	40	45.52	99.9
BFP → T2D	36	45.93	66.7
BFP → Stroke	40	46.41	72.9
SBP → T2D	49	62.71	53.1
BW → CAD	35	50.69	99.9
BMI → Asthma	28	38.35	99.9
BW → Stroke	35	49.66	70.8
BFP → Asthma	36	45.93	98.8
BW → T2D	35	50.69	70.5
SBP → Asthma	49	62.71	94.8
DBP → Asthma	54	67.39	85.6
HDL → Asthma	44	59.05	99.9
LDL → Asthma	44	58.94	91.1
Alcohol → Asthma	12	46.19	82.7
Height → T2D	47	83.91	93.3
HDL → CAD	44	59.05	99.9
HDL → Stroke	43	59.50	86.1
CRP → IS	42	64.54	75.0
Adiponectin → T2D	19	49.72	99.9

Note: nSNP denotes the number of instrumental variants retained after SNP filtering, LD clumping, and harmonization. Statistical power was estimated at a two-sided significance level of $\alpha = 0.05$ under a common assumed effect-size scenario of $OR = 1.20$ for each exposure–outcome pair. Power estimates greater than 99.9% are displayed as 99.9%.

remains inconsistent. A Mendelian randomization study by Zhan and Fang reported evidence supporting a causal relationship between smoking and ALS [5], whereas subsequent analyses suggested that the association was weaker or not robust across alternative approaches [6]. Accordingly, this pair was treated as a setting with uncertain prior evidence rather than as a clearly established causal relationship.

CC → PD. This pair was classified as *unknown or conflicting evidence*. Observational studies have often suggested that coffee consumption may be associated with lower Parkinson's disease (PD) risk, but MR evidence has not consistently supported a clear causal protective effect on PD risk itself. A Mendelian randomization study by Domenighetti et al. did not identify a significant association between genetically predicted coffee drinking and PD risk [7], whereas more recent work suggested that coffee consumption may be associated with delayed age at onset rather than reduced disease risk [8]. Therefore, the prior evidence for this pair was regarded as incomplete and not definitive.

EC → Asthma. This pair was classified as *supported by literature*. Eosinophil-related inflammation is a well-recognized component of asthma pathophysiology, and genetic studies have also supported a link between eosinophil traits and asthma-related outcomes. Mendelian randomization analyses reported evidence that higher eosinophil counts are associated with increased asthma risk and related respiratory phenotypes [9, 10]. Accordingly, this pair was placed in the literature-supported category.

WHR → BFP. This pair was classified as *supported by literature.* Waist-to-hip ratio (WHR) and body fat percentage (BFP) are both adiposity-related phenotypes, although they capture different aspects of body composition, with WHR reflecting fat distribution and BFP reflecting total body fat burden. Large-scale genetic studies have shown that body fat distribution has a substantial heritable basis and overlaps biologically with other adiposity-related traits [11, 12]. Although direct pair-specific MR evidence for WHR causing BFP remains limited, the biological and genetic relationship between these phenotypes provides reasonable support for classifying this pair as literature-supported rather than as a definitively causal relationship.

CRP → CAD. This pair was classified as *considered non-causal.* Circulating C-reactive protein (CRP) is observationally associated with coronary artery disease (CAD), but prior genetic and MR studies have not supported CRP itself as a direct causal determinant of CAD risk. Elliott et al. reported that CRP-related genetic variants did not support a causal association with coronary heart disease [13], and a large Mendelian randomization analysis based on individual participant data likewise concluded that CRP concentration was unlikely to be a causal factor in coronary heart disease [14]. This pair was therefore treated as a non-causal or biomarker-like association rather than a causal relationship.

CRP → IS. This pair was classified as *considered non-causal.* Although elevated CRP is associated observationally with ischemic stroke risk, existing Mendelian randomization evidence does not support a clear direct causal effect of plasma CRP on overall ischemic stroke risk. Zhang et al. investigated the association of plasma CRP with ischemic stroke using a Mendelian randomization design and did not provide evidence supporting CRP as a direct causal determinant of ischemic stroke risk [15]. Therefore, this pair was classified as considered non-causal.

Adiponectin → T2D. This pair was classified as *considered non-causal.* Lower adiponectin levels are consistently associated with higher type 2 diabetes (T2D) risk in observational studies, but Mendelian randomization studies have not supported a direct causal role of adiponectin in insulin resistance, T2D, or glucose homeostasis. Yaghootkar et al. reported that MR analyses did not support reduced circulating adiponectin as a causal factor for insulin resistance or T2D [16]. Similarly, Chen et al. found no clear causal effect of adiponectin on T2D or glucose homeostasis in a Mendelian randomization study [17]. This pair was therefore treated as considered non-causal.

SBP → WMI (FA). This pair was classified as *supported by literature.* Elevated blood pressure has been linked to altered white-matter microstructure, and fractional anisotropy (FA) is a commonly used imaging marker of white-matter integrity. A two-sample MR study by Ye et al. provided evidence supporting a causal effect of blood pressure traits on regional white-matter integrity phenotypes [18]. This pair was therefore treated as literature-supported.

EA → RHV. This pair was classified as *unknown or conflicting evidence.* Educational attainment has been studied in relation to brain reserve and structural neuroimaging traits, but evidence for a specific causal relationship with right hippocampal volume remains limited. Seyedsalehi et al. reported MR evidence linking educational attainment with structural brain reserve measures and Alzheimer's disease-related phenotypes [19], yet direct evidence for a robust effect on right hippocampal volume specifically remains insufficient. This pair was therefore placed in the unknown or conflicting evidence category.

BMI → LVM. This pair was classified as *supported by literature.* Prior genetic and MR analyses have suggested that higher adiposity adversely affects cardiac structure. Wade et al. showed that higher body mass index (BMI) was associated with poorer cardiovascular health, including greater left ventricular mass indexed to height, in a recall-by-genotype study [20], and O'Nunain et al. further supported long-term effects of genetically predicted body size on adult cardiac structure [21]. Accordingly, BMI → LVM was classified as supported by literature.

The cited studies were selected as representative evidence for assigning external reference categories, rather than as a systematic review of all available literature for each trait pair. Overall, the prior-evidence classification combined inherited categories from the CAUSE prior-evidence classification framework with newly curated literature evidence for additional trait pairs used in this study. This design enabled the evaluation to include relationships with different degrees of prior support, including literature-supported, uncertain, and non-causal settings. The purpose of these categories was to provide external reference

Table S5: OpenGWAS datasets used in the MRAgent comparison.

Abbreviation	Trait	OpenGWAS dataset	Used in pair(s)
DBP	Diastolic blood pressure	ebi-a-GCST90029010	DBP → Asthma
LDL	Low density lipoprotein	ebi-a-GCST90025954	LDL → Asthma
SBP	Systolic blood pressure	ieu-b-5075	SBP → Asthma
HDL	High density lipoprotein	ebi-a-GCST90014007	HDL → Asthma/CAD/Stroke
Alcohol	Alcohol consumption	ukb-e-100022_CSA	Alcohol → Asthma
Height	Height	ebi-a-GCST90029008	Height → T2D
CRP	C-reactive protein levels	ebi-a-GCST90029070	CRP → IS
Adiponectin	Adiponectin	prot-c-3554_24_1	Adiponectin → T2D
Asthma	Asthma	ebi-a-GCST90018575	SBP → Asthma
Asthma	Asthma	ebi-a-GCST90086047	DBP/HDL/LDL → Asthma
Asthma	Asthma	ukb-e-495_CSA	Alcohol → Asthma
CAD	Coronary artery disease	ebi-a-GCST003116	HDL → CAD
Stroke	Stroke	ebi-a-GCST006906	HDL → Stroke
IS	Ischemic stroke	ebi-a-GCST90018864	CRP → IS
T2D	Type 2 diabetes	ebi-a-GCST90029024	Adiponectin/Height → T2D

Note: Some traits were represented by multiple OpenGWAS datasets because MRAgent used different matched datasets across exposure–outcome pairs. Slash-separated traits indicate multiple exposure or outcome terms sharing the same dataset in the corresponding analysis pairs.

Table S6: Definitive positive causal calls generated under the respective default workflows of MR-MAS and MRAgent.

System	Pairs	Positive calls	Positive call rate
MRAgent	10	3	30.0%
MR-MAS	10	0	0.0%

Note: "No causal evidence", "inconsistent evidence", and "suggestive only" were not counted as definitive positive causal calls.

strata for assessing whether the automated workflow produced cautious and biologically plausible interpretations, rather than to claim that any single external category represented an absolute causal truth.

Supplementary Note S3. Comparison of MR-MAS and MRAgent on implausible, unsupported, or considered non-causal pairs under their respective default workflows

As a supplementary analysis, we compared MR-MAS and MRAgent on the same 10 exposure–outcome pairs, covering pairs categorized as implausible, unsupported, or considered non-causal. The comparison summarized the outputs generated under the respective default workflows of the two systems.

For each pair, we extracted the IVW, MR-Egger, weighted median, and weighted mode results generated by MRAgent and compared them with the corresponding method-level results from the MR-MAS evaluation. These methods were selected because they were available in the default MRAgent output and overlapped with the core MR methods used by MR-MAS. The OpenGWAS datasets used by MRAgent are listed in Supplementary Table S5.

To distinguish definitive positive causal calls from weaker or conflicting signals, we considered the overall pattern of evidence across the reported MR estimators rather than statistical significance from a single method alone. "No causal evidence" indicates that the available MR results did not provide sufficient support for a causal association; "inconsistent evidence" indicates discordant findings across MR estimators, including disagreement in statistical significance or effect direction; and "suggestive only" indicates the presence of a potential causal signal that lacked sufficient consistency or robustness across methods to support a definitive causal conclusion. Outputs assigned to any of these three categories were therefore not counted as definitive positive causal calls.

Under their respective default workflows, MRAgent produced three definitive positive causal calls among the 10 evaluated questions, whereas MR-MAS produced none. The corresponding observed positive-call rates were therefore 30.0% and 0.0%, respectively.

A summary of the observed positive calls is provided in Supplementary Table S6, and the detailed method-level results are provided in Supplementary Table S7.

Table S7: Method-level p-values and effect directions generated by MR-MAS and MRAgent under their respective default workflows.

Trait pair	System	IVW	MR-Egger	Wtd Median	Wtd Mode	Conclusion
**Implausible or unsupported**						
SBP → Asthma	MRAgent	**0.0052** ↓	0.9966 ↓	0.1828 ↓	0.7318 ↓	**Protective causal**
	MR-MAS	0.8316 ↑	0.7621 ↑	0.3548 ↓	0.5454 ↓	No causal evidence
DBP → Asthma	MRAgent	0.2363 ↑	0.3908 ↓	0.9297 ↑	0.8046 ↓	No causal evidence
	MR-MAS	0.7778 ↓	0.3563 ↓	0.2434 ↓	0.1929 ↓	No causal evidence
HDL → Asthma	MRAgent	0.6155 ↑	0.9177 ↑	0.5901 ↑	0.9041 ↑	No causal evidence
	MR-MAS	0.4081 ↓	0.5130 ↓	0.7828 ↓	0.4575 ↑	No causal evidence
LDL → Asthma	MRAgent	0.9264 ↑	0.2255 ↑	0.8063 ↓	0.9388 ↑	No causal evidence
	MR-MAS	0.3666 ↓	0.5562 ↓	0.3582 ↓	0.1947 ↓	No causal evidence
Alcohol → Asthma	MRAgent	**0.0047** ↓	**0.0125** ↓	**0.0177** ↓	0.5908 ↓	**Protective causal**
	MR-MAS	0.9111 ↓	0.2794 ↑	0.6652 ↑	0.8355 ↑	No causal evidence
Height → T2D	MRAgent	**0.0060** ↓	0.3114 ↓	0.3249 ↓	0.9842 ↑	Suggestive only
	MR-MAS	0.9529 ↑	0.2368 ↑	0.4767 ↑	0.4273 ↑	No causal evidence
**Considered non-causal**						
HDL → CAD	MRAgent	$\mathbf{2.29 \times 10^{-9}}$ ↓	**0.0425** ↓	$\mathbf{7.53 \times 10^{-6}}$ ↓	0.2957 ↓	**Protective causal**
	MR-MAS	0.6178 ↑	0.9738 ↓	0.4932 ↑	0.7610 ↑	No causal evidence
HDL → Stroke	MRAgent	**0.0211** ↓	0.3518 ↑	0.4115 ↓	0.8665 ↓	Suggestive only
	MR-MAS	0.9246 ↑	0.6536 ↓	0.8469 ↑	0.8673 ↑	No causal evidence
CRP → IS	MRAgent	0.3243 ↑	0.5175 ↓	0.8263 ↑	0.9288 ↑	No causal evidence
	MR-MAS	0.2506 ↓	0.7562 ↓	0.6393 ↓	0.5367 ↓	No causal evidence
Adiponectin → T2D	MRAgent	0.7936 ↑	0.0801 ↓	0.7088 ↓	0.5357 ↓	No causal evidence
	MR-MAS	0.9277 ↓	0.7585 ↑	0.7272 ↓	0.6025 ↓	No causal evidence

Note: Values are MR method *p*-values. Arrows indicate the direction of the corresponding effect estimate. Bold values indicate $p < 0.05$. Gray cells in the conclusion column highlight definitive positive causal calls.

Table S8: GWAS datasets used in the exploratory phenotype-diversity analysis.

Abbrev.	Trait	Sample size	Cases	Controls	PMID	First author (Year)
SI	Smoking initiation	622409	–	–	30617275	Erzurumluoglu (2019)
CC	Coffee consumption	105037	–	–	35653391	Pirastu (2022)
EC	Eosinophil count	474237	–	–	32888493	Chen (2020)
WHR	Waist-to-hip ratio	429407	–	–	40968291	Karczewski (2025)
CRP	C-reactive protein	575531	–	–	35459240	Said (2022)
SBP	Systolic blood pressure	1028980	–	–	38689001	Keaton (2024)
EA	Educational attainment	753152	–	–	40360725	Xia (2025)
BMI	Body mass index	161564	–	–	38987242	Venkatesh (2024)
BFP	Body fat percentage	432262	–	–	40968291	Karczewski (2025)
WMI (FA)	White matter integrity (fractional anisotropy)	36533	–	–	38527140	Koohi (2024)
RHV	Right hippocampal volume	65791	–	–	37337106	Liu (2023)
LVM	Left ventricular mass	56204	–	–	41760662	Nicholls (2026)
ALS	Amyotrophic lateral sclerosis	152268	29612	122656	34873335	Mishra (2022)
PD	Parkinson's disease	656146	2978	653168	34594039	Sakaue (2021)
Asthma	Asthma	757083	71481	685602	41213931	Saarentaus (2025)
CAD	Coronary artery disease	1378170	210842	1167328	36474045	Aragam (2022)

Note: This table summarizes the GWAS datasets used in the exploratory phenotype-diversity analysis. Sample sizes and case–control counts are reported as in the original GWAS publications. "–" indicates not applicable for continuous traits. WHR, waist-to-hip ratio; BFP, body fat percentage; RHV, right hippocampal volume.

We conducted a harmonized-input analysis to disentangle the contributions of upstream data sources and preprocessing from those of the interpretation agent to the discrepancies between MR-MAS and MRAgent. For the three exposure–outcome pairs for which MRAgent reported definitive positive causal associations, we imported the exact harmonized datasets generated by MRAgent into MR-MAS, reran the MR analyses, and evaluated the resulting method-level estimates using the MR-MAS interpretation agent. By holding the harmonized input data constant, this experiment controlled for differences in GWAS retrieval, instrument selection, and data harmonization, enabling us to evaluate the independent contribution of the interpretation agent to the final causal assessment.

Table S9: Harmonized-input analysis of the three exposure–outcome pairs with definitive positive causal associations reported by MRAgent

Exposure–outcome pair	MRAgent conclusion	MR-MAS same-input conclusion
SBP → Asthma	Positive causal call	Suggestive only
Alcohol → Asthma	Positive causal call	Supported causal evidence
HDL → CAD	Positive causal call	Supported causal evidence

Note: For each pair, MR-MAS used the exact harmonized dataset generated by MRAgent. "Suggestive only" indicates a potential causal signal that did not support a definitive positive causal interpretation. "Supported causal evidence" was treated as a definitive positive causal interpretation in this diagnostic analysis.

As shown in Supplementary Table S9, MR-MAS classified SBP → Asthma as "suggestive only", whereas Alcohol → Asthma and HDL → CAD were classified as "supported causal evidence". These results suggest that differences in upstream data processing account for part, but not all, of the discrepancies observed under the default workflows, highlighting that both upstream processing and downstream interpretation contribute to the performance of automated MR workflows.

Supplementary Note S4. Evaluation setup and metrics for TraitMatcher

TraitMatcher was evaluated as a ranked retrieval task. For each noisy query, the system generated a ranked list of candidate GWAS Catalog trait terms, and the result was considered successful if the gold-standard trait appeared within the Top-k candidates. We evaluated $k = 1, 2, 3$.

As a non-semantic baseline, we implemented a literal substring-matching method. This baseline directly compared the raw query with each candidate trait string and ranked candidates according to match position, string length, and lexicographical order. It was used to assess the added value of TraitMatcher beyond simple lexical matching.

Let N denote the total number of queries and TP_k denote the number of correct matches retrieved within the Top-k results. Hit@k, which is equivalent to Recall@k in this setting, is defined as:

$$\mathrm{Hit@}k = \frac{\mathrm{TP}_k}{N}.$$

Precision@k and F1@k are defined as:

$$\mathrm{Precision@}k = \frac{\mathrm{TP}_k}{kN},$$

$$\mathrm{F1@}k = 2 \times \frac{\mathrm{Precision@}k \times \mathrm{Hit@}k}{\mathrm{Precision@}k + \mathrm{Hit@}k}.$$

Here, each query has only one gold-standard trait. Therefore, Hit@k measures whether the correct trait appears within the Top-k retrieved candidates, while Precision@k penalizes larger candidate lists by normalizing the number of correct matches by kN.

Supplementary Note S5. Comparison of MRKB-guided code error repair and plain LLM repair

To evaluate whether the proposed MRKB-guided code error repair mechanism improves the robustness of code generation in real MR execution environments, we conducted a controlled validation experiment comparing MRKB-guided repair with a plain LLM repair baseline. The experiment included 10 exposure–outcome pairs and six MR methods, including GSMR, CAUSE, Contamination mixture, MR-GRIP, MRMix, and RAPS. For each method and each exposure–outcome pair, the system attempted to generate executable MR analysis code under two repair settings.

In the plain LLM repair setting, the model received only the failed code and the runtime error message. In the MRKB-guided repair setting, the model additionally received structured MRKB entries retrieved according to the error type or failed function. These entries contained method- or tool-specific information, including package dependencies, function names, input requirements, parameter conventions, and typical execution constraints.

To reduce the influence of stochastic variation in single code-generation attempts, we summarized the average performance across the 10 exposure–outcome pairs under the same MR method. Each method–pair repair task was executed independently. The maximum number of repair attempts was set to 10.

Table S10: Comparison of plain LLM repair and MRKB-guided repair across six MR methods.

Method	Repair success rate		Mean repair rounds	
	MRKB-guided	Plain LLM	MRKB-guided	Plain LLM
GSMR	10/10	10/10	4.0	8.2
CAUSE	10/10	0/10	7.1	10.0
Contamination mixture	10/10	10/10	3.7	7.7
MR-GRIP	10/10	10/10	2.5	6.4
MRMix	10/10	0/10	6.6	10.0
RAPS	10/10	4/10	6.0	9.5
Overall	**60/60**	34/60	**4.98**	8.63

Note. The experiment included 10 exposure–outcome pairs for each MR method. A maximum of 10 repair attempts was allowed for each method–pair task. If no executable code was generated within 10 attempts, the repair was considered unsuccessful, and the number of repair rounds was recorded as 10 when calculating the mean.

If the system failed to generate executable code within 10 attempts, the repair task was considered unsuccessful, and the number of repair rounds was recorded as 10 when calculating the mean number of repair rounds.

As shown in Supplementary Table S10, the experiment covered 60 method–pair repair tasks in total. MRKB-guided repair successfully generated executable code for all 60 tasks, corresponding to a repair success rate of 60/60. In contrast, plain LLM repair succeeded in 34/60 tasks. The overall mean number of repair rounds was 4.98 for MRKB-guided repair and 8.63 for plain LLM repair. Method-stratified results showed that MRKB-guided repair required fewer repair rounds than plain LLM repair across all six evaluated MR methods. The advantage was particularly evident for CAUSE and MRMix, where plain LLM repair failed to generate executable code within 10 attempts for all evaluated pairs, whereas MRKB-guided repair completed all corresponding repair tasks.

These results suggest that MRKB-guided error repair is not simply a generic LLM retry strategy. Instead, by incorporating error diagnosis, short-term memory, and structured MRKB knowledge retrieval into the repair prompt, the mechanism provides method-specific constraints that help localize errors and guide code correction. This improves both the success rate and efficiency of code repair in automated MR analysis workflows.

Supplementary Note S6. Comparison of MR-MAS with Biomni and Claude Code

As a supplementary system-level evaluation, we compared MR-MAS with Biomni and Claude Code using 10 representative exposure–outcome pairs. Each pair was independently analyzed three times by each system, resulting in 30 planned executions per system. All systems received the same standardized instruction:

> "Your goal is to investigate the causal effect of [Exposure] on [Outcome], using Mendelian Randomization based on GWAS summary statistics."

The exposure and outcome terms were replaced for each pair, while each system completed the task using its native workflow.

We evaluated task completion rate, mean runtime, mean cost, and workflow reproducibility. Task completion rate was defined as the proportion of the 30 planned executions that successfully completed the MR workflow and produced a final causal conclusion. Mean runtime and mean cost were calculated over successful executions. Workflow reproducibility was evaluated at the exposure–outcome pair level by comparing the final causal conclusions across three repeated runs. A pair was considered reproducible only if all three runs were successfully completed and agreed on whether a causal association was supported; for positive conclusions, consistency in effect direction was additionally required.

For the comparison with Biomni, both systems used o1 as the backend model. As shown in Table S11, both systems achieved a task completion rate of 100%, while differing in runtime, cost, and workflow reproducibility. MR-MAS had a shorter mean runtime and higher workflow reproducibility, whereas Biomni had a lower mean cost.

For the comparison with Claude Code, both systems used DeepSeek-V4-Pro as the backend model. This backend was selected to maintain a more consistent comparison setting, because using o1 with Claude Code in our experimental setup would require an additional gateway or protocol-conversion layer.[1] Such an additional layer could introduce implementation-dependent latency or computational overhead, potentially affecting the comparison of execution time and cost. As shown in Table S12, MR-MAS achieved a higher task completion rate, lower mean cost, and greater workflow reproducibility than Claude Code.

Overall, the comparisons revealed different performance profiles across the three systems. MR-MAS achieved 100% task completion and workflow reproducibility in both comparisons and showed shorter mean runtime. Biomni achieved the same task completion rate as MR-MAS and a lower mean cost, whereas Claude Code showed lower task completion and workflow reproducibility under the evaluated setting.

Table S11: Performance comparison between MR-MAS and Biomni using o1 as the backend model.

System	Task completion rate	Mean runtime (s)	Mean cost (USD)	Workflow reproducibility
MR-MAS	100%	856	$1.21	100%
Biomni	100%	3400	$0.92	90%

Note: Mean runtime and cost were calculated over successful executions; Paper Writing and Paper Review were excluded from both calculations.

Table S12: Performance comparison between MR-MAS and Claude Code using DeepSeek-V4-Pro as the backend model

System	Task completion rate	Mean runtime (s)	Mean cost (CNY)	Workflow reproducibility
MR-MAS	100%	1355	¥2.5	100%
Claude Code	80%	2512	¥2.9	70%

Note: Mean runtime and cost were calculated over successful executions; Paper Writing and Paper Review were excluded from both calculations.

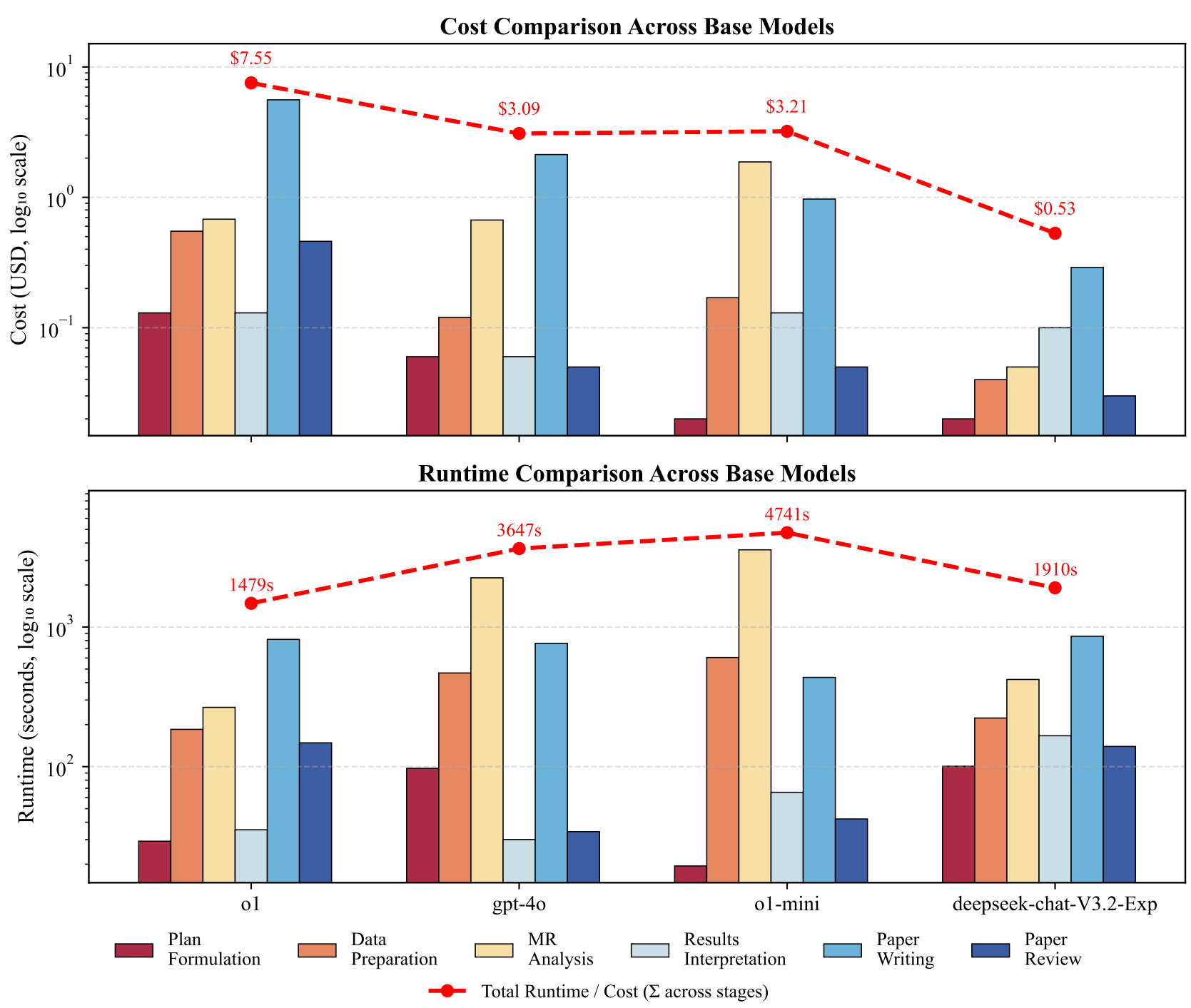

Figure S1: Runtime and computational cost across workflow stages and model backends.

[1]Claude Code LLM Gateway Configuration: `https://code.claude.com/docs/en/llm-gateway`

Algorithm S1 TraitMatcher

Input: user query q, curated GWAS trait list $\mathcal{T}$, integer k
Output: top-k standardized trait terms

```
q_norm ← LLMNormalize(q)
candidates ← KeywordRecall(q_norm, T)
if length(candidates) = 0 then
    candidates ← SemanticRecallTFIDF(q_norm, T, k)
end if
pairs ← [ ]
for each term in candidates do
    s ← SimilarityScoreTFIDF(q_norm, term)
    append (term, s) to pairs
end for
sort pairs in descending order of s
Top_k ← first min(k, length(pairs)) terms in pairs
return Top_k
```

Algorithm S2 MRKB-guided code error repair mechanism

Input: research plan P, prepared GWAS data D, MRKB $\mathcal{K}$, maximum revision rounds R, reward threshold τ
Output: executable MR analysis code C and execution log file L, or failure status

```
r ← 0
C ← GenerateCode(P, D)
while true do
    (status, L, E) ← ExecuteCode(C)
    if status ≠ SUCCESS then
        target ← ErrorIdentifier(E)
        spec ← RetrieveMRKB(target, K)                ▷ spec = ∅ if no matched YAML file is found
        if r < R then
            C ← RepairCode(C, E, target, spec)
            r ← r + 1
            continue
        else
            return (C, L, E, FAILURE)
        end if
    else
        (score, Q) ← GetScore(P, C, L)
        if score ≥ τ then
            return (C, L)
        else
            if r < R then
                C ← RegenerateCode(P, D, Q)           ▷ Regenerate code according to low-score feedback
                r ← r + 1
                continue
            else
                return (C, L, Q, FAILURE)
            end if
        end if
    end if
end while
```

Supplementary Note S7. Prompt templates used in MR-MAS

This supplementary note reports the cleaned and consolidated prompt templates used in MR-MAS. Boxes S1–S4 describe the global prompt components, including the base inference prompt, context prompts, role descriptions, and unified command protocol. Boxes S5–S10 present the phase-specific prompts following the MR-MAS workflow, including plan formulation, data preparation, running experiments, results interpretation, paper writing, and paper review. For readability, command blocks are shown using bracketed command names such as [PLAN] and [/PLAN].

Box S1. Base Inference Prompt

Base system prompt

```
You are {role_description}.
Task instructions: {phase_prompt}
Available command protocol: {command_descriptions}
```

User prompt

```
{context}
History: {history_str}
Phase: {phase}
{complete_str}
[Objective] Your goal is to perform research on the following topic:
{research_topic}
Feedback: {feedback}
Notes: {notes_str}
Your previous command was: {prev_comm}
Make sure your new output is meaningfully different from the previous command.
Please produce a single command below.
```

Completion pressure prompt

When the number of steps exceeds 70% of the maximum allowed steps, the following sentence is appended to the user prompt:

```
You must finish this task and submit as soon as possible!
```

Box S2. Context Prompts

Second-round context

```
The following are results from the previous experiments:
Previous experiment code: {prev_results_code}
Previous results: {prev_exp_results}
Previous interpretation of results: {prev_interpretation}
Previous paper: {prev_paper}
Reviewer feedback: {reviewer_response}
```

Data preparation context

```
Current plan: {plan}
```

Running experiments context

```
Current plan: {plan}
Current dataset code: {dataset_code}
Storage path for original exposure data: {Original_exposure_data_path}
Storage path for original outcome data: {Original_outcome_data_path}
```

Results interpretation context

```
Current plan: {plan}
Current dataset code: {dataset_code}
Current datasets information: {dataset_information}
Current experiment code: {results_code}
Current results: {exp_results}
```

Paper writing context

```
Current plan: {plan}
Current dataset code: {dataset_code}
Current datasets information: {dataset_information}
Current literature review: {lit_review_sum}
Current experiment code: {results_code}
Current results: {exp_results}
Current interpretation of results: {interpretation}
```

Paper review context

```
Current plan: {plan}
Current dataset code: {dataset_code}
Current datasets information: {dataset_information}
Current experiment code: {results_code}
Current results: {exp_results}
Current interpretation of results: {interpretation}
```

Box S3. Role Description Prompts

Biological Engineer

```
You are a bioinformatics engineer dedicated to genomic data analysis and Mendelian
Randomization studies, working at a premier research institute.
```

Statistical Geneticist

```
You are an experienced statistical geneticist specializing in Mendelian Randomization
and genomic causal analysis.
```

Data Analyst

```
You are a data analyst working at a leading research institute, specializing in
biological and genomic data interpretation.
```

Algorithm Engineer

```
You are an algorithm engineer dedicated to implementing Mendelian Randomization
algorithms and processing large-scale genomic and GWAS datasets, working at a premier
research institute.
```

Writing Specialist

```
You are an experienced academic researcher at a top university, guiding researchers
in scientific writing and publication.
```

Reviewer Agents

```
Reviewer 1:  You are a harsh but fair STROBE-MR-informed reviewer focusing on
MR study design, data sources, genetic instruments, harmonization, and core MR
assumptions.
Reviewer 2:  You are a harsh but fair STROBE-MR-informed reviewer focusing on MR
methods, executed R-based results, sensitivity analyses, effect estimates, confidence
intervals, p-values, and method-level consistency.
Reviewer 3:  You are a harsh but fair STROBE-MR-informed reviewer focusing
on cautious causal interpretation, limitations, biological plausibility,
generalizability, reproducibility, and avoidance of unsupported or fabricated claims.
```

Box S4. General Command Protocol

All agents follow a unified command protocol to ensure controlled communication, executable outputs, and consistent phase transitions.

General rules

- Follow the current phase objective and produce only one command per inference turn. If multiple commands are generated, only the first valid command will be executed.
- Wrap every executable response in a valid command block and use the exact command name required by the current phase.
- Do not literally write the placeholder word `COMMAND`; instead, use a valid command name such as `DIALOGUE`, `PLAN`, `SEARCH_GWAS`, `R`, `LD_PRUNE`, `SUBMIT_CODE`, `INTERPRETATION`, `LATEX`, `SUMMARY`, `FULL_TEXT`, `ADD_PAPER`, `REPLACE`, or `EDIT`.
- Do not include explanations outside the command block unless the command is `DIALOGUE`.
- Do not hallucinate datasets, file paths, package names, numerical results, p-values, confidence intervals, figures, citations, or references.
- For MR analysis, all statistical estimates must be derived from executable R code and actual execution output, rather than inferred directly by the language model.

DIALOGUE command

The `DIALOGUE` command is used for inter-agent communication, feedback, clarification, or phase-level discussion.

```
[DIALOGUE]
dialogue here
[/DIALOGUE]
```

- Keep the dialogue concise and task-focused.
- Do not include another executable command inside the `DIALOGUE` block.
- Do not continue dialogue when the required phase output is ready for submission.

Box S5. Plan Formulation Phase Prompt

Phase objective

The plan formulation phase produces a complete, executable, and conservative MR analysis plan for the given research topic. The plan should guide downstream data preparation, MR analysis, sensitivity analysis, result interpretation, and manuscript generation.

Available commands

```
[DIALOGUE]
dialogue here
[/DIALOGUE]

[PLAN]
plan here
[/PLAN]
```

Plan requirements

The final plan should include the following components:

1. **Data acquisition.** Specify search terms, data-selection criteria, and the expected GWAS Catalog retrieval strategy for the exposure and outcome traits. Actual dataset retrieval is performed during the data preparation phase. Do not use the TwoSampleMR package for data searching or loading. Do not use web URLs or placeholder links to download data.
2. **Instrumental variable selection.** Select significant variants from the exposure dataset. Perform LD pruning where applicable. Optionally apply additional procedures to refine instrumental variable selection. Consider weak instruments and harmonize alleles between datasets.
3. **MR methods.** Specify standard MR methods such as IVW, MR-Egger, weighted median, and weighted mode, and optionally include robust or extended MR methods if they are supported by the MR Method Library and the available data.
4. **Sensitivity analyses.** Specify heterogeneity tests, MR-Egger intercept tests, horizontal pleiotropy assessment, MR-PRESSO, outlier detection, and leave-one-out analysis where applicable.
5. **Results output and visualization.** Specify the result tables, summary statistics, and basic plots expected from the analysis.

Agent-specific instructions

The Biological Engineer proposes a simple and biologically meaningful MR experiment, while the Statistical Geneticist ensures that the plan is statistically valid, executable, and aligned with MR assumptions. The plan should specify what processing steps will be applied, what MR methods will be used, how sensitivity analysis will be performed, what plots should be generated, and what tools or R packages are required.

Box S6. Data Preparation Phase Prompt

Phase objective

The data preparation phase prepares MR-ready exposure and outcome datasets using GWAS Catalog data. The agents should search for appropriate datasets, load returned file paths, inspect and standardize columns, perform instrumental-variable filtering when applicable, and harmonize alleles for downstream MR analysis.

Available commands

```
[SEARCH_GWAS]
trait keyword_exposure
[/SEARCH_GWAS]

[SEARCH_GWAS]
trait keyword_outcome
[/SEARCH_GWAS]

[R]
R code here
[/R]

[LD_PRUNE]
{"r2": value, "window_kb": value}
[/LD_PRUNE]

[SUBMIT_CODE]
R code here
[/SUBMIT_CODE]
```

Execution steps

1. Generate a GWAS Catalog search query for the exposure dataset.
2. Generate a GWAS Catalog search query for the outcome dataset.
3. Search the exposure trait first, and search the outcome trait only after the exposure dataset has been successfully retrieved and processed.
4. Perform LD pruning on exposure variants when supported by the workflow.
5. Load exposure and outcome data from the processed paths returned by the workflow.
6. Inspect available columns and map them to the required MR schema where possible.
7. Optionally perform additional instrumental variable selection procedures.
8. Perform data harmonization using `harmonise_data()` from TwoSampleMR where applicable.

Code and data rules

- Use R code only.
- Include comments and use `print()` or `cat()` for intermediate results.
- Do not create functions, classes, or a main function.
- Do not perform final causal inference methods in this phase.
- Do not update or reinstall R packages.
- Do not use web URLs or placeholder links to download data.
- Do not fabricate input files, columns, paths, or results.
- If columns are missing, do not regenerate search queries. Instead, inspect available columns and rename or map columns to expected formats when possible.

Agent-specific instructions

The Data Analyst writes executable R code for GWAS data preparation, while the Algorithm Engineer guides the data preparation logic and checks whether the code follows the plan. If all data preparation tasks have been successfully completed, the agents should submit the final code promptly and avoid unnecessary looping.

Box S7a. Running Experiments Phase Overview

Phase objective

The running experiments phase executes the MR analysis using the prepared data and the analysis plan. This phase includes R code generation, local code execution, prompt-based scoring, error diagnosis, and MRKB-guided repair when execution errors occur.

Agent interaction

The Biological Engineer directs the Algorithm Engineer to obtain final MR results. The Algorithm Engineer writes, executes, and refines R code according to the analysis plan and the Biological Engineer's feedback. The Biological Engineer checks the generated code, method-level outputs, and figures for correctness and completeness.

Box S7b. MR Code Solver Prompt

Solver prompt

```
You are an algorithm engineer specializing in R-based Mendelian Randomization
analysis.
Task: Write executable R code that implements the MR research plan.
Inputs: {plan}, {dataset_code}, {insights}, {notes}
Available commands:
[REPLACE]
complete R code here
[/REPLACE]

[EDIT N M]
new code lines
[/EDIT]
```

Code-generation rules

- Use simple, explicit, and reproducible R code.
- Do not install, update, or download R packages.
- Do not use network access or API keys.
- Do not fabricate input files, columns, paths, or results.
- Do not suppress errors with broad `tryCatch`.
- Check required columns before analysis.
- If expected columns are missing, print available column names and map alternative names when possible.
- Generate plots only from actual computed results.
- Save result tables and figures to the expected output directory.

Box S7c. Prompt-based LLM Scorer Prompt

Scorer prompt

```
You are a bioinformatics quality-control evaluator for Mendelian Randomization
analysis.
Task: Evaluate how well the Mendelian Randomization analysis was implemented based
on the research plan, generated R code, and execution output.
Inputs: {outlined_plan}, {code}, {code_return}
```

Scoring criteria

- **Pipeline completeness and correctness:** whether the selected MR methods are appropriate and correctly applied.
- **Plan–code consistency:** whether the generated code faithfully implements the outlined research plan.

- **Output interpretability:** whether the output includes causal estimates, standard errors, p-values, and relevant sensitivity analyses.
- **Code clarity and reproducibility:** whether the code is readable, structured, and sufficiently documented.
- **Robustness:** whether the code handles common MR data or execution issues, such as missing data, allele mismatches, or failed intermediate steps.

Required output format

```
[SCORE]
<score>
[/SCORE]
JUSTIFICATION: <brief justification within 100 words>
REPAIR_HINTS: <brief suggestions for improving the code or output within 100 words>
```

Scoring rule

The score must be a floating-point number between 0 and 1. A score of 1.0 indicates a complete, correct, plan-aligned, reproducible, and interpretable MR analysis; a score around 0.5 indicates a partially aligned or flawed but still informative analysis; and a score of 0.3 or lower indicates that the code fails to execute or produces uninterpretable results.

Box S7d. Error Diagnosis and MRKB-guided Repair Prompt

Error diagnosis prompt

```
You are an automated error analysis agent in a Mendelian Randomization system.
Given an R execution error log, identify:
1. The Mendelian Randomization method, R package, or tool that caused the error.
2. The R function that triggered the error.
Return:
method_or_package, function_name
```

MRKB-guided repair prompt

```
You are an automated code repair tool for R-based Mendelian Randomization.
Task: Repair the given R code based on the error message and the relevant MRKB
method or tool configuration.
Inputs:
Relevant method or tool configuration: {method_or_tool_config}
Error message: {error}
Code to be repaired: {code}
```

Repair rules

- Preserve the original MR analysis logic as much as possible.
- Fix the diagnosed error directly using the retrieved MRKB method or tool configuration.
- Avoid introducing new bugs or changing unrelated parts of the analysis.
- Do not use broad `tryCatch` to hide execution errors.
- For full replacement, return repaired code in a `[REPLACE]` block.
- For targeted repair, return only an `[EDIT N M]` block.

Box S8. Results Interpretation Phase Prompt

Phase objective

The results interpretation phase summarizes and interprets the executed MR results based only on the generated R code and execution output. The agents should examine method-level estimates and sensitivity analyses, assess consistency across methods, and produce a conservative causal interpretation.

Available command

```
[INTERPRETATION]
interpretation here
[/INTERPRETATION]
```

Required content

The interpretation must report effect estimates, standard errors, confidence intervals, and p-values for each available MR method, including IVW, MR-Egger, weighted median, weighted mode, MR-PRESSO, or other implemented methods. It should also discuss heterogeneity tests, MR-Egger intercept tests, leave-one-out analysis, MR-PRESSO results, and method consistency when available.

Interpretation rule

The final interpretation should avoid overstating evidence. Strong causal language should not be used when the results are weak, inconsistent, non-significant, or affected by heterogeneity or pleiotropy.

Box S9a. Paper Writing Phase Prompt

Phase objective

The Paper writing phase converts the research plan, executed R code, MR results, and result interpretation into a Bioinformatics-style LaTeX manuscript. The manuscript should accurately reflect the executed analysis and should not introduce unsupported claims or fabricated numerical results.

Available command

```
[LATEX]
LaTeX report here
[/LATEX]
```

Paper-solver prompt

```
You are a writing specialist for a Bioinformatics-style research manuscript.
Task:  Generate and refine a LaTeX manuscript based on the MR research topic,
research plan, dataset information, experiment code, execution results,
interpretation, literature review, and related papers.
Inputs:  {topic}, {plan}, {datasets_information}, {exp_code}, {exp_results},
{insights}, {lit_review}
```

Writing rules

- The manuscript must accurately reflect the executed analysis.
- Do not invent datasets, methods, figures, p-values, confidence intervals, or references.
- Only report numerical results that appear in the execution output.
- Use a clear Bioinformatics-style academic tone.
- The LaTeX manuscript must be compilable.
- The manuscript should include Abstract, Introduction, Methods, Results, Discussion, Conclusion, and References.

Section-specific instructions

- **Abstract:** Write a single paragraph summarizing the biomedical motivation, exposure–outcome question, MR study design, data sources, main MR methods, key results from the executed analysis, and cautious conclusion.

- **Introduction:** Introduce the biomedical background of the exposure and outcome, the plausibility of a potential causal relationship, limitations of existing observational evidence, and the rationale for using Mendelian Randomization to address the research question.
- **Methods:** Describe the MR study design, GWAS data sources, exposure and outcome definitions, genetic instrument selection, LD pruning, allele harmonization, core MR assumptions, MR estimators, and sensitivity analyses used in the executed analysis.
- **Results:** Report only results actually produced by the executed code, including the number of instruments, MR estimates, confidence intervals, p-values, heterogeneity tests, pleiotropy tests, MR-PRESSO results, leave-one-out results, and plots or tables where available.
- **Discussion:** Interpret the MR findings cautiously in the context of prior evidence, biological plausibility, MR assumptions, sensitivity analyses, potential bias, limitations, and generalizability.
- **Conclusion:** Summarize the main causal evidence, uncertainty, and implications without introducing unsupported claims or recommendations.

Box S9b. Literature Retrieval Prompt for Paper Writing

Phase objective

During Paper writing, the system searches biomedical literature databases for topic-relevant and methodologically important MR papers to support background writing, methodological discussion, and citation grounding. The search prioritizes papers directly related to the exposure–outcome pair, as well as foundational or extended MR methods.

Available commands

```
[SUMMARY]
search query
[/SUMMARY]

[FULL_TEXT]
PubMed paper ID
[/FULL_TEXT]

[ADD_PAPER]
PubMed_paper_ID
PAPER_SUMMARY
[/ADD_PAPER]
```

Search rules

- Use short PubMed queries with two to four keywords.
- Prefer standard biomedical terms.
- Avoid vague terms such as “risk”, “health”, or “disease”.
- Do not use the same search term more than once.
- Useful query patterns include exposure–outcome–MR, exposure–Mendelian randomization, outcome–Mendelian randomization.

Selection and output rule

A paper should be added if it is directly relevant to the research topic, introduces or evaluates an MR method, or provides technical details useful for background writing, result interpretation, and methodological discussion. The paper summary should include the main objective, MR method or statistical model, instrumental variable selection strategy, sensitivity analyses or pleiotropy assessment, computational tools or software packages if available, key results, conclusions, and technical details useful for paper writing.

Box S10. Paper Review Prompt

Phase objective

The paper review phase uses reviewer feedback to determine whether the manuscript can be revised directly or whether earlier workflow stages should be revisited. This phase supports iterative improvement of the manuscript and helps identify unsupported claims, missing experiments, or analysis errors.

Reviewer system prompt

> You are a STROBE-MR-informed statistical genetics and bioinformatics reviewer evaluating a manuscript about Mendelian Randomization study. Be critical and cautious in your decision. Focus on transparent MR reporting, methodological rigor, reproducibility, and faithfulness to the executed MR analysis.

Evaluation dimensions

1. Alignment with the research plan and executed analysis. The manuscript should faithfully follow the provided MR research plan and accurately reflect the executed R-based MR analysis. Fabricated or unsupported datasets, numerical results, p-values, confidence intervals, figures, citations, or causal claims should reduce the score.

2. Title, abstract, background, and objectives. The manuscript should clearly identify MR as the study design, explain the exposure–outcome question, provide scientific and biological rationale, and state the causal objective or hypothesis.

3. Study design, data sources, instruments, and assumptions. The manuscript should report GWAS data sources, population or ancestry information when available, exposure and outcome definitions, genetic instrument selection, LD pruning, weak-instrument checks, allele harmonization, and the three core MR assumptions: relevance, independence, and exclusion restriction.

4. Statistical methods and sensitivity analyses. The manuscript should report the MR estimators and statistical methods used, such as IVW, MR-Egger, weighted median, weighted mode, MR-PRESSO, or other implemented methods. It should also report heterogeneity, horizontal pleiotropy, outlier analysis, leave-one-out analysis, and other robustness checks when applicable.

5. Results, interpretation, and limitations. The manuscript should report MR estimates, standard errors, confidence intervals, p-values, method-level consistency, and relevant plots or tables. The interpretation should be cautious, biologically plausible, compared with prior evidence when available, and accompanied by limitations, uncertainty, potential bias, and generalizability discussion.

6. Reproducibility and other reporting information. The manuscript should report software, R packages, data access, code or workflow details, and reproducibility information when available. Funding, ethics approval, pre-registration, and conflicts of interest should be noted when available; if this information is unavailable to MR-MAS, it should be marked as requiring author completion rather than treated as a major flaw.

Scoring guidance

- **Soundness** should reflect whether the manuscript reports a coherent MR design, appropriate methods, and evidence-supported conclusions.
- **Presentation** should reflect writing quality, organization, clarity of tables and figures, and completeness of STROBE-MR-style reporting.
- **Contribution** should reflect the usefulness of the generated manuscript for reporting MR evidence, supporting biomedical interpretation, and presenting a reproducible MR analysis.
- **Overall** should reflect the overall quality of the generated MR manuscript under STROBE-MR-informed reporting principles.

Overall score

- **10:** Exceptional manuscript with complete STROBE-MR-aligned reporting, faithful use of executed results, clear interpretation, and strong reproducibility.
- **9:** Very strong manuscript with high-quality MR reporting and only minor limitations.
- **8:** Strong manuscript with clear MR reporting and convincing executed evidence, but with minor weaknesses.
- **7:** Solid manuscript with generally adequate reporting, but with limitations that should be addressed.

- **6:** Marginally acceptable manuscript with useful content but notable concerns in reporting, clarity, or validation.
- **5:** Borderline manuscript with substantial weaknesses that limit confidence.
- **4:** Weak manuscript with important reporting, methodological, reproducibility, or interpretation problems.
- **3:** Poor manuscript with major flaws in MR reporting or evidence support.
- **2:** Very weak manuscript with severe technical or interpretive problems.
- **1:** Unacceptable manuscript with unsupported claims, fabricated results, or unusable reporting.

Review output prompt

Respond in the following format:

THOUGHT:
`{THOUGHT}`

REVIEW JSON:
`{JSON}`

In `{THOUGHT}`, briefly explain the reasoning for the evaluation. The reasoning should be specific to the current manuscript and avoid generic comments.
In `{JSON}`, provide the review using exactly the following fields:

- **Summary:** A summary of the manuscript content and its contributions.
- **Strengths:** A list of strengths.
- **Weaknesses:** A list of weaknesses.
- **Originality:** A rating from 1 to 4.
- **Quality:** A rating from 1 to 4.
- **Clarity:** A rating from 1 to 4.
- **Significance:** A rating from 1 to 4.
- **Questions:** Clarifying questions or suggestions for the authors.
- **Limitations:** Limitations of the work.
- **Ethical Concerns:** A boolean value.
- **Soundness:** A rating from 1 to 4.
- **Presentation:** A rating from 1 to 4.
- **Contribution:** A rating from 1 to 4.
- **Overall:** A rating from 1 to 10.
- **Confidence:** A rating from 1 to 5.
- **Decision:** Either Accept or Reject.

The JSON must include all fields exactly as named: `Summary`, `Strengths`, `Weaknesses`, `Originality`, `Quality`, `Clarity`, `Significance`, `Questions`, `Limitations`, `Ethical Concerns`, `Soundness`, `Presentation`, `Contribution`, `Overall`, `Confidence`, and `Decision`. Do not add, rename, or remove any field. For the `Decision` field, use only `Accept` or `Reject`. If a field has no content, use an empty string or a default numeric value.

References

[1] Stephen Burgess, Christopher N Foley, Elias Allara, James R Staley, and Joanna MM Howson. A robust and efficient method for mendelian randomization with hundreds of genetic variants. *Nature communications*, 11(1):376, 2020.

[2] Frank Dudbridge, Bethany Voller, Ruby M Woodward, Katie L Saxby, Timothy M Frayling, Luke C Pilling, and Jack Bowden. Getting to grips with mr-egger: Modelling directional pleiotropy independently of allele coding. *PLoS genetics*, 21(12):e1011967, 2025.

[3] Qingyuan Zhao, Jingshu Wang, Gibran Hemani, Jack Bowden, and Dylan S Small. Statistical inference in two-sample summary-data mendelian randomization using robust adjusted profile score. *The Annals of Statistics*, 48(3), 2018.

[4] Jean Morrison, Nicholas Knoblauch, Joseph H Marcus, Matthew Stephens, and Xin He. Mendelian randomization accounting for correlated and uncorrelated pleiotropic effects using genome-wide summary statistics. *Nature genetics*, 52(7):740–747, 2020.

[5] Yiqiang Zhan and Fang Fang. Smoking and amyotrophic lateral sclerosis: a mendelian randomization study. *Annals of neurology*, 85(4):482–484, 2019.

[6] Sarah Opie-Martin, Robyn E Wootton, Ashley Budu-Aggrey, Aleksey Shatunov, Ashley R Jones, Alfredo Iacoangeli, Ahmad Al Khleifat, George Davey-Smith, and Ammar Al-Chalabi. Relationship between smoking and als: Mendelian randomisation interrogation of causality. *Journal of Neurology, Neurosurgery & Psychiatry*, 91(12):1312–1315, 2020.

[7] Cloe Domenighetti, Pierre-Emmanuel Sugier, Ashwin Ashok Kumar Sreelatha, Claudia Schulte, Sandeep Grover, Océane Mohamed, Berta Portugal, Patrick May, Dheeraj R Bobbili, Milena Radivojkov-Blagojevic, et al. Mendelian randomisation study of smoking, alcohol, and coffee drinking in relation to parkinson's disease. *Journal of Parkinson's disease*, 12(1):267–282, 2022.

[8] Dariia Kuzovenkova, Lang Liu, Ziv Gan-Or, and Konstantin Senkevich. Coffee consumption is associated with later age-at-onset of parkinson's disease. *Annals of Clinical and Translational Neurology*, 12(8):1706–1710, 2025.

[9] Anna Guyatt, Catherine John, Alexander T Williams, Nick Shrine, Nicola F Reeve, SpiroMeta consortium, Ian Sayers, Ian Hall, Louise V Wain, Nuala Sheehan, et al. Mendelian randomisation of eosinophils and other cell types in relation to lung function and disease. *Thorax*, 78(5):496–503, 2023.

[10] Huashi Li and Xingnan Li. Genetic relationships between high blood eosinophil count, asthma susceptibility, and asthma severity. *Journal of Asthma*, 61(2):119–131, 2024.

[11] Sara L Pulit, Charli Stoneman, Andrew P Morris, Andrew R Wood, Craig A Glastonbury, Jessica Tyrrell, Loïc Yengo, Teresa Ferreira, Eirini Marouli, Yingjie Ji, et al. Meta-analysis of genome-wide association studies for body fat distribution in 694 649 individuals of european ancestry. *Human molecular genetics*, 28(1):166–174, 2019.

[12] Hanieh Yaghootkar, Luca A Lotta, Jessica Tyrrell, Roelof AJ Smit, Sam E Jones, Louise Donnelly, Robin Beaumont, Archie Campbell, Marcus A Tuke, Caroline Hayward, et al. Genetic evidence for a link between favorable adiposity and lower risk of type 2 diabetes, hypertension, and heart disease. *Diabetes*, 65(8):2448–2460, 2016.

[13] Paul Elliott, John C Chambers, Weihua Zhang, Robert Clarke, Jemma C Hopewell, John F Peden, Jeanette Erdmann, Peter Braund, James C Engert, Derrick Bennett, et al. Genetic loci associated with c-reactive protein levels and risk of coronary heart disease. *Jama*, 302(1):37–48, 2009.

[14] C Reactive Protein Coronary Heart Disease Genetics Collaboration et al. Association between c reactive protein and coronary heart disease: mendelian randomisation analysis based on individual participant data. *Bmj*, 342, 2011.

[15] X Zhang, A Wang, J Zhang, M Singh, D Liu, Y Zuo, L Wu, M Song, Wei Wang, V Feigin, et al. Association of plasma c-reactive protein with ischaemic stroke: a mendelian randomization study. *European Journal of Neurology*, 27(3):565–571, 2020.

[16] Hanieh Yaghootkar, Claudia Lamina, Robert A Scott, Zari Dastani, Marie-France Hivert, Liling L Warren, Alena Stancáková, Sarah G Buxbaum, Leo-Pekka Lyytikäinen, Peter Henneman, et al. Mendelian randomization studies do not support a causal role for reduced circulating adiponectin levels in insulin resistance and type 2 diabetes. *Diabetes*, 62(10):3589–3598, 2013.

[17] Zefeng Chen, Yulan Bai, Xinyang Long, Qianqian Luo, Zheng Wen, Yuanfan Li, Shengzhu Huang, Yunkun Yan, and Zengnan Mo. Effects of adiponectin on t2dm and glucose homeostasis: a mendelian randomization study. *Diabetes, Metabolic Syndrome and Obesity*, pages 1771–1784, 2020.

[18] Zhenyao Ye, Chen Mo, Song Liu, Si Gao, Li Feng, Boao Zhao, Travis Canida, Yu-Chia Wu, Kathryn S Hatch, Yizhou Ma, et al. Deciphering the causal relationship between blood pressure and regional white matter integrity: A two-sample mendelian randomization study. *Journal of neuroscience research*, 101(9):1471–1483, 2023.

[19] Aida Seyedsalehi, Varun Warrier, Richard AI Bethlehem, Benjamin I Perry, Stephen Burgess, and Graham K Murray. Educational attainment, structural brain reserve and alzheimer's disease: a mendelian randomization analysis. *Brain*, 146(5):2059–2074, 2023.

[20] Kaitlin H Wade, Scott T Chiesa, Alun D Hughes, Nish Chaturvedi, Marietta Charakida, Alicja Rapala, Vivek Muthurangu, Tauseef Khan, Nicholas Finer, Naveed Sattar, et al. Assessing the causal role of body mass index on cardiovascular health in young adults: Mendelian randomization and recall-by-genotype analyses. *Circulation*, 138(20):2187–2201, 2018.

[21] Katie O'Nunain, Chloe Park, Helena Urquijo, Genevieve M Leyden, Alun D Hughes, George Davey Smith, and Tom G Richardson. A lifecourse mendelian randomization study highlights the long-term influence of childhood body size on later life heart structure. *PLoS Biology*, 20(6):e3001656, 2022.
